# Prospection, Perseverance, and Insight in Sequential Behavior

**DOI:** 10.1016/j.neuron.2018.08.018

**Published:** 2018-09-05

**Authors:** Nils Kolling, Jacqueline Scholl, Adam Chekroud, Hailey A. Trier, Matthew F.S. Rushworth

**Affiliations:** 1 Department of Experimental Psychology, Nuffield Department of Clinical Neurosciences, University of Oxford, Oxford, UK; 2 Oxford Centre of Human Brain Activity, Nuffield Department of Clinical Neurosciences, University of Oxford, Oxford, UK; 3 Centre for Functional MRI of the Brain (MRI), Nuffield Department of Clinical Neurosciences, University of Oxford, Oxford, UK; 4 Department of Psychiatry, School of Medicine, Yale University, New Haven, CT, USA

**Keywords:** dorsal anterior cortex, reward, decision making, human neuroimaging, sequential behaviors, computational cognitive neuroscience

## Abstract

Real-world decisions have benefits occurring only later and dependent on additional decisions taken in the interim. We investigated this in a novel decision-making task in humans (n = 76) while measuring brain activity with fMRI (n = 24). Modeling revealed that participants computed the *prospective* value of decisions: they planned their future behavior taking into account how their decisions might affect which states they would encounter and how they themselves might respond in these states. They considered their own likely future behavioral biases (e.g., failure to adapt to changes in prospective value) and avoided situations in which they might be prone to such biases. Three neural networks in adjacent medial frontal regions were linked to distinct components of prospective decision making: activity in dorsal anterior cingulate cortex, area 8 m/9, and perigenual anterior cingulate cortex reflected prospective value, anticipated changes in prospective value, and the degree to which prospective value influenced decisions.

## Highlights

• People use prospective information about future state when making sequential choices
• People have insight into their future biases and pre-emptively avoid key situations
• Distinct cingulate-prefrontal interactions encode prospective and myopic value
• Perigenual cingulate motivates perseveration in sequential behavior

## Teaser

Kolling and Scholl et al. examined extended sequential decision-making problems. Participants used prospective information to inform even initial decision planning several steps ahead. Dorsal cingulate activity signaled prospective value while perigenual cingulate activity motivated decision sequence performance.

## Introduction

Many decisions depend on the values of objects or goods that might be chosen and the neural mechanisms mediating those decisions are increasingly well understood (Abitbol et al., 2015, Howard et al., 2016, Hunt and Hayden, 2017, Rudebeck and Murray, 2014, Rushworth et al., 2011). However, in other cases, our choices are determined by quite different factors. It may be important to consider not only the short-term, but also the longer-term implications of a choice, or in other words, the choice’s “prospective value.” This crucially depends on one’s own future behavioral strategy—i.e., how one will respond to changes in the environment that are not yet known fully at the time of the initial decision. Because such changes may in turn be due to one’s own future behavior, studying prospective decision making in the laboratory is difficult and has received comparatively little attention.

**Figure 1:**
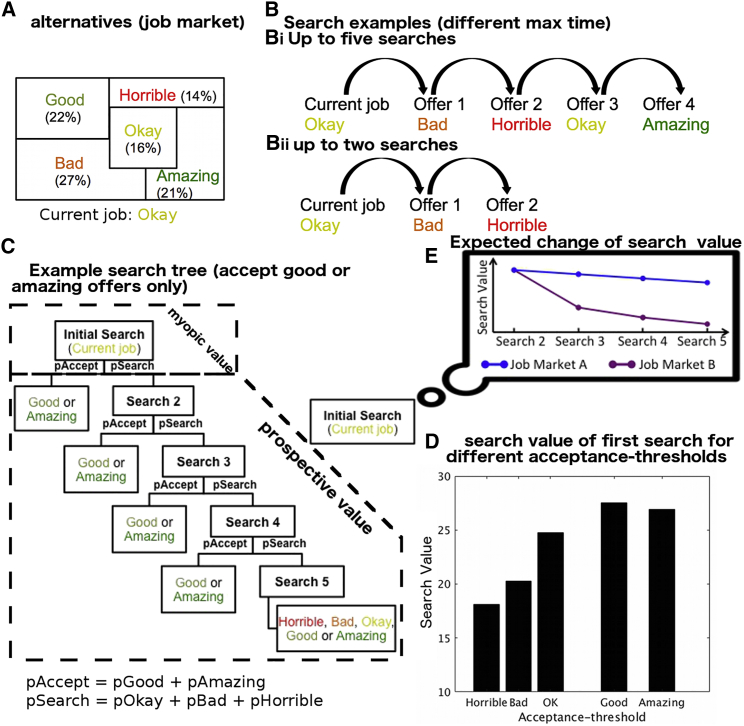
Prospective Decision Making (A) A job market has different jobs (horrible to amazing) with specific odds (percentage and patch size). (B) Decision making should depend not just on the average value of the options, but instead one should take into account the search horizon—how many weeks one could afford to search. If the search horizon is long (Bi), the job seeker might reject inferior offers, hoping for the dream job. However, if the search horizon is shorter (Bii), one might have to accept an inferior job for fear of a worse offer in the end. (C) The ideal solution for deciding whether one should search for a new job or not, is to simulate a decision tree of the value of all possible outcomes and their probabilities, given what kinds of jobs one would accept, at a given acceptance threshold (Figure S1A illustrates a numeric example). In this example, the acceptance threshold is to accept jobs that are at least “good.” (D) Computing the overall search value of the decision tree in this way is then repeated for each possible acceptance threshold (see also Figure S1B). For example, the first bar in the histogram illustrates the search value, for a given time horizon, of setting the acceptance threshold at “horrible” and above; any job offer is accepted. The next bar illustrates the search value of setting the acceptance threshold at “bad” and above and so on. In this example, search value is maximized by setting the acceptance threshold at “good” and above. If the decision maker is too “unfussy” and accepts any offer (left) or too fussy and is only prepared to accept “amazing” jobs (right), then search value is lower. (E) The search value can decrease at different rates as the number of future opportunities decreases. This is because environments can differ in their statistical properties, some of which are more affected by reduced search horizons. For example, if the variance of an environment is very high, its value will fall more quickly as receiving unlikely but very good outcomes becomes unlikely. Knowing about the decreases in search value and how quickly value changes might affect the initial decision a person takes.

For example, deciding whether to quit one’s job and enter the job market depends not only on the quality of one’s job, but also the current state of the job market (Figure 1A). If one can only accept or reject a single offer at any given time, it is important to consider the maximum amount of time one can look for a job (time or search horizon; Figure 1B). If, for example, the job seeker can wait ten weeks with relatively minor waiting costs, it might be worth rejecting inferior offers while holding out for the dream job (Figure 1C). If, however, the search horizon is restricted, one might have to accept an inferior job for fear of ending up with an even worse offer or because one can simply not wait any longer. In other words, a job seeker should not solely consider the average of all available jobs (“myopic value”), as this would fail to reflect the opportunities they will have to reject specific interim offers in the hope of getting a future offer that is better than the current average (Figure 1D). In other words, the prospective value of an environment is more than just its average or myopic value (even if opportunities are sampled with replacement). This effect should become even more pronounced if one has a longer search horizon: the prospective value of the job market is even higher if you can reject bad offers and search for longer. A long search horizon is particularly beneficial when there is variability in the quality of job offers; there is more to be gained from waiting for the best offer if that offer is far from the mean value of jobs.

In summary, the overall value of the environment (sometimes called “search value”) can be decomposed into myopic and prospective components. The myopic component corresponds to the average benefits that might immediately follow a decision while prospective value corresponds to future benefits that might accrue over the longer term by taking a particular choice now. While the neural correlates of myopic value have previously been investigated (Kolling et al., 2012, Stoll et al., 2016) here we focus on prospective value. Delay discounting (Kable and Glimcher, 2007), spatial planning (Kaplan et al., 2017), or simpler fixed decision sequences (Kolling et al., 2014, Symmonds et al., 2010) on the other hand do not require self-determined planning of multiple and sequential choices nor the use of prospective value.

In some ways the scenario resembles the classic secretary problem (Costa and Averbeck, 2015, Ferguson, 1989) that considers how many secretaries an administrator should interview to learn enough about the quality of secretaries to make an optimal job offer. However, secretary problems are not just about decision making but also about optimal information sampling. By contrast, we are examining a pure decision situation in which no learning is necessary because all information is explicitly shown.

Importantly in such scenarios, to judge the prospective value of an initial decision, an agent needs insight into the decision strategy it will use in the future when it encounters options. We propose that the decision strategy can be derived from a simple decision rule inspired by prey-encounter models (Blanchard and Hayden, 2014, Stephens and Krebs, 1986). The future decision strategy determines the probabilities for accepting options and when as the search horizon diminishes (Figures 1D and 1E). For this, the model assumes a threshold for future decisions: a minimum value at which an option should be accepted. With such a model an overall expected value for any environment can be easily computed for every possible threshold (Figure S1; STAR Methods). Without a future decision rule, it is not possible to determine the complete value of the environment over the longer term. The threshold has important implications: if it is too low, one misses the opportunity to wait for something better; too high and one may pass up a good opportunity. It is also of note that as fewer and fewer searches remain, the value of searching decreases (Figure 1E). This is for the same reason as discussed above when comparing environments in which many or few searches were available initially (Figure 1B).

## Results

**Figure 2:**
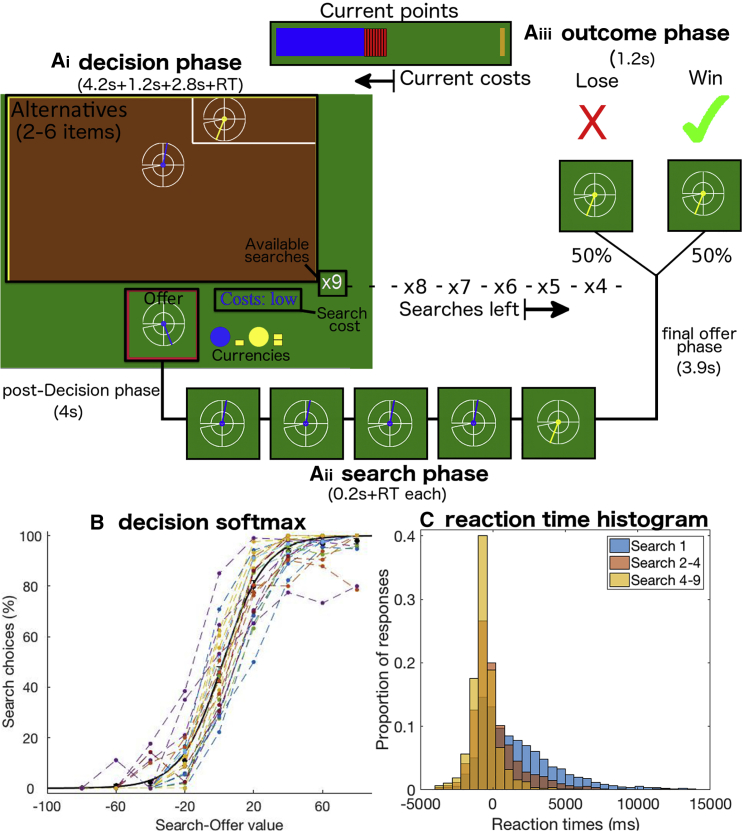
Example Trial (A) On each trial, participants decided whether to accept an offer shown at the bottom of the screen. (Ai) The offer’s reward magnitude was indicated by the position and color of the dial (yellow indicated twice the reward magnitude as blue; clockwise dial position indicated number of points between 5 and 160). If a participant rejected the offer, then they searched for an alternative (between two and six in each environment); they received a new offer drawn from the search environment (“alternatives”) at the top of the screen. The probability was indicated by size of the area occupied (here the blue and yellow alternatives had probabilities of 90% and 10%). Participants were also shown the search horizon (nine possible searches in this example) and how much it would cost to do so (search cost is low [six points] here but could also be “none” or “high” [12 points]). The order of feature presentation was randomized. (Aii) If a participant chose to search, then they potentially embarked on a sequence of decisions to accept or reject each new offer drawn from the alternatives. Each time they searched, they received a new offer from the alternatives, the cost was subtracted (top), and the number of available searches was reduced by one (display of alternatives remained the same because alternatives were sampled with replacement). The search phase ended either with an accept decision or there were no searches left. (Aiii) Only in 50% of trials (tick) were rewards actually received in the subsequent outcome phase (crosses signaled losses). (B) Acceptance decisions varied with the difference between search value derived from the computational decision-tree model (Figure 1C, which reflected the magnitude of alternatives, their probabilities, costs, search horizon) and offer value. Black circles: participants’ mean data binned by value difference; black line: simulated choices using a computational model fitted on all participants together; colored lines: each participant’s actual choices (binned). (C) Histograms of demeaned reaction times for initial and subsequent decisions. The initial decisions took longer than subsequent decisions even though participants had several seconds to examine options before being cued to make the first response.

We developed a novel task to measure how humans decide to initiate a course of behavior entailing a sequence of “search decisions” and how they subsequently negotiated the sequence. We collected participants in the lab with fMRI (n = 24) and online via the internet (n = 51). On each trial of the task (160 for the fMRI sample), participants tried to win as many points (equal to money) as possible. Participants were shown an “offer,” a symbol (a dial) denoting the number of points to be won. They chose to accept or reject the offer (Figure 2Ai). If they accepted the offer they moved on to the outcome phase to discover whether they had won or lost (50% probability; Figure 2Aiii). The inclusion of probabilistic reward delivery meant that it was possible, even with fMRI, to separate neural activity related to the choice from subsequent neural activity related to offer receipt.

**Figure 3:**
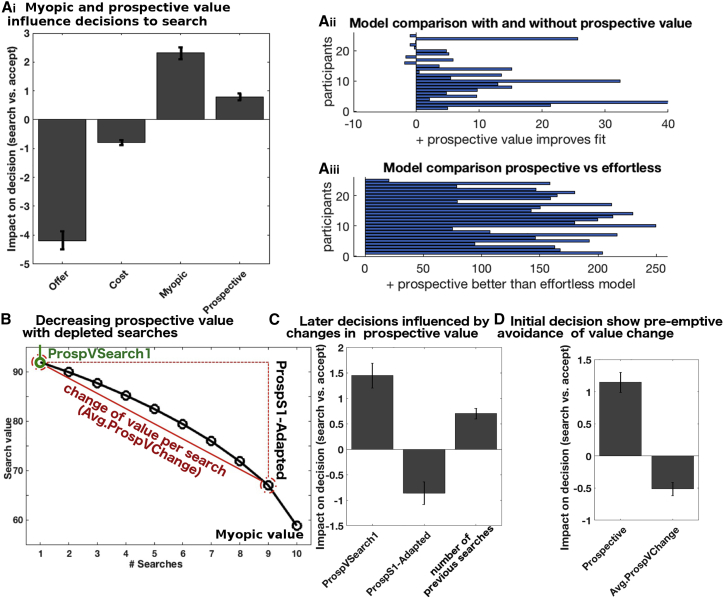
Behavioral Results (A) Search decisions (Ai) were more likely as offer value and search costs decreased and as the model-derived myopic and prospective values (which together constituted search value) increased (all p < 0.00001). (Aii) Model comparison of model with prospective value against one with several non-prospective heuristics (bGLM1e2) but without prospective value (bGLM1e; summed AIC difference: 175.7; figure shows average AIC differences for individual participants, across all searches). See also Figure S2 for other types of heuristics. (Aiii) Model comparison prospective vs effortless strategy. Similarly, the prospective value model (bGLM1a) was better than a model using a “planless” or “effortless strategy” (bGLM1d; summed AIC difference:3929). (B) Example of search value decrease with depleting searches (here trial started with 10 searches). The decrease is due to diminishing prospective value, which goes to zero for the last search, resulting in the only value being myopic. Prospective value at search one is called “ProspVSearch1” and changes between this and current search is “ProspS1-Adapted”. The average prospective value change (Avg.ProspVChange) is the difference between the initial search value and the last search with any prospective value divided by the number of available searches. (C) Participants adjusted their prospective value estimates (bGLM3a; ProspS1-Adapted, p < 0.001). Searching also became more likely with more previous searches within a trial even when controlling for all resulting changes in value. (D) At the first search in a trial, people avoided initiating searches in sequences with rapidly declining prospective value, suggesting insight (bGLM5a, p < 0.001).

In contrast, if participants rejected the offer they received a new offer from the set of alternatives (the “environment”; Figure 2Aii). We refer to this choice as “searching” (Kolling et al., 2012). For each alternative, participants saw the reward magnitude (dial) and the probability of it being drawn if they searched (indicated by the proportion of the area in the environment occupied by an alternative). Participants were shown how much they would have to pay to search (“search cost”) and how many more times they could search on a given trial (search horizon or “available searches”). Each time participants searched, the cost was subtracted from the points that they had accumulated so far and the number of available searches decreased by one. This search phase continued until participants had exhausted the total number of searches or accepted an offer they had encountered while searching. We used a parametric-model-based experimental design, which has several advantages. First, we can disentangle several distinct aspects of value behaviorally and neurally using multiple regressions. This is particularly important for dissociating prospective from myopic search value. Second, we can control for other possible confounds such as the difficulty of a decision itself (Kolling et al., 2016a, Kolling et al., 2016b). An important addition in the online study was a debrief questionnaire at the end of the experiment asking participants about their strategies and probing their insight. Task details for the online sample differed slightly (see STAR Methods), but the key task features remained constant and behavioral performance was comparable (cf. Figures 3 and S5).

### Participants Make Decisions Prospectively

When deciding whether to search or accept the current offer, participants should consider the set of alternatives, search costs, remaining search horizon, and the future accept-search decisions they would make for subsequent offers (Figure 1). In other words, they should employ the information contained in the decision trees, derived from prey-encounter models (Figure 1C). The computational model (STAR Methods; Figure S1) used considers all possible acceptance thresholds (i.e., minimum offer value that will be accepted) in a trial. For each acceptance threshold, the model builds a complete decision tree of all possible states (a state is defined by the offer and number of searches used), how likely these states are to be encountered, and their values to calculate the expected value of searching (“search value”). To determine the actual value of searching in a trial, the model simply picks the acceptance threshold that yields the highest search value (Figure 1D). We found that participants behaved as expected from the model: they modulated their decisions to search or accept the current offer as a function of the search value (Figure 2B).

As described in the Introduction, search value can be split into an immediate myopic value (sum of all the alternatives’ magnitudes × probabilities) and a prospective value. The decision-tree model was used to compute the latter, but it is not necessary for the former (as it is the straight-forward average of all values in the environment). General linear model (GLM) analyses were used to examine the influence of the two types of value on participants’ decision making (precise details of each behavioral GLM [bGLM] and fMRI GLM [fGLM] are provided in STAR Methods and in Table S1). Participants’ decisions reflected both myopic and prospective values (bGLM1a; myopic: t_24_ = 11.07, p = 6 × 10^−11^; prospective: t_24_ = 6.64, p = 7 × 10^−7^; Figure 3Ai). In several control analyses, we examined whether, instead of computing prospective value, participants used simpler non-prospective heuristics; i.e., we wanted to rule out the possibility that participants did not actually plan ahead. In short, we found strong evidence that participants’ decisions are better explained by models incorporating prospective elements. As we discuss later, this does not mean that participants perform a tree search that is identical to that employed by our computational model in every last detail. Participants may well approximate a tree-search using some heuristic. What we can rule out, however, is that participants’ decisions are informed solely by non-prospective heuristics.

**Figure 4:**
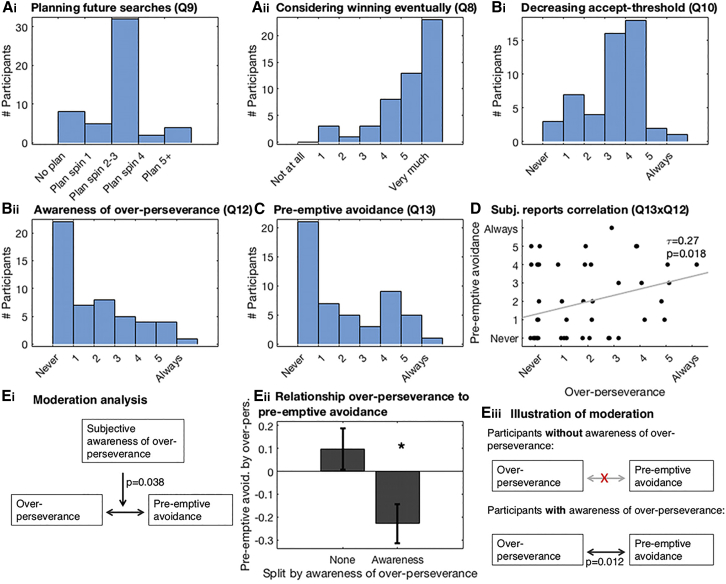
Self-Report Questionnaires Collected in the Online Sample, Relating to Behaviors in Figure 3 (A) Histogram of debrief questionnaire scores confirmed participants (n = 51) planned their future searches (Ai). They did this taking into account the prospective element of being able to reject potentially undesirable offers until eventually obtaining a desirable offer (Aii). (B) They also reported that, over the course of a trial, they adapted their search strategy by decreasing their acceptance threshold (Bi). Some were also aware that they nevertheless sometimes over-persevered (Bii). (C) Participants reported that, to avoid over-perseverance later in the trial, they sometimes pre-emptively avoided trials. (D) Indeed, there was a correlation between the subjective reports of over-perseverance and the subjective report of pre-emptive avoidance (non-parametric correlation: Kendall’s tau = 0.27, p = 0.018). (E) The relationship between the behavioral measures of over-perseverance and pre-emptive avoidance (from our regression model [bGLM3a, Figure 3C and bGLM5a, Figure 3D]) was moderated by explicit subjective awareness (Ei: t_43_ = −2.14, p = 0.038, n = 47). (Eii) To illustrate this moderation effect, participants were split into those who reported no awareness that they ever over-persevered and those who did report that they at least sometimes over-persevered. Only in participants that were aware that they over-persevered was there a relationship between how much they over-persevered (regression weight for number of previous searches, Figure 3C) and how much pre-emptive avoidance they showed (regression weight for Avg.ProspVChange, Figure 3D): the more biased a participants was to over-persevere, the more likely they were to pre-emptively avoid trials with potentially large decreases in search value, requiring adaptation of search strategy (t_43_ = −2.63, p = 0.012). The same result is also illustrated with box-and-arrow diagrams (Eiii). See STAR Methods for full wording of the debrief questionnaire questions.

The first set of non-prospective heuristic that we tested included regressors for simple properties of the environment that could be used to make decisions (bGLM1e). We compared this model with an identical model that additionally included the prospective value term and found that the latter model provided a better account of the data (Figure 3Aii, summed AIC difference: 175.7; see Figure S2 for results of a related analysis using an alternative set of simple properties). Next, we considered a second alternative strategy that participants might use instead of prospection—i.e., instead of building a mental (tree) model of what they might do in the future: participants could use an almost effortless decision rule to reject any offer that is not the best possible offer until they have only one search left. At that point they should accept anything that is not the worst offer (bGLM1d). However, we find that our model that included prospective value (bGLM1a) explains behavior better (Figure 3Aiii). The participants’ subjective reports given during debriefing also indicated that they used mental simulation of what might happen if they searched and of what they might do if they encountered various opportunities (Figure 4A).

In the prospective tree-search model, as fewer and fewer searches remain on a trial after continued engagement, prospective value decreases (Figure 1E); when only one search remains, the search value becomes equivalent to the myopic value (Figure 3B). Importantly, this means that, when the search horizon has shrunk, you should accept some options that you should have rejected earlier. To test whether participants flexibly adapted search value, as predicted from the model, as they progressed through a trial, we split prospective value at the current search into two components: its initial value at the start of the sequence and how much it had decreased by the current search in the sequence (bGLM3a). We discovered that participants’ behavior over the course of a sequence of searches was sensitive to decreases in prospective value (Figure 3C): the more prospective value decreased, the less likely participants were to search compared to what would have been expected had they continued to use the initial prospective value without adapting it (bGLM3a, regression weight for the change in prospective value: t_24_ = −3.93, p < 0.001). Similarly, participants reported during debriefing that they adapted their estimate of prospective value (i.e., that they accepted offers later in a trial that they would have rejected early on, Figure 4Bi).

Although participants were clearly making adjustments to the way they made decisions during the sequence, surprisingly they made those adjustments despite responding faster and faster as they progressed through each sequence (Figures 2C and S3A, main effect of first search on reaction time [RT]: t_24_ = 7.39, p < 0.001). This suggests that participants did not re-compute the decision tree online in the same manner as initially after every search. One way participants might do this is by exploiting the fact that prospective value decays over the course of searching in an approximately exponential manner (Figures 3B and S3B). Indeed, we found that participants’ reaction times later in sequences were better described by search values based on the exponential approximation rather than true prospective values (Figure S2E).

### Planning Using Future Decision Tendencies

So far, we have considered all decisions within sequences together. However, incorporating future possibilities when making an initial search decision is only useful if you later take advantage of the possibilities when they arise. For example, as discussed in the previous section, an initial decision that is based on a prospective value estimate that decreases in an adaptive manner as the sequence progresses is only valuable if you do indeed actually go on to adjust your behavior. If participants possess insight and realize that their behavior later in the sequence may be subject to non-optimal biases, then such insight should affect how their initial decision to search is taken. In other words, when they compute the prospective value in a sequence they need to plan in a way that is consistent with the behavior they themselves are likely to produce in the future.

One notable bias exhibited by participants was a tendency to over-persevere; participants tended to search more than was optimal given the model-derived estimate of search value (appropriately adapted for progress through the sequence as the search horizon shrunk). Specifically, participants were more likely to search yet again as a function of how often they had already searched earlier in the sequence (Figure 3C, bGLM3a, t_24_ = 6.81, p < 0.001). Again, we found further support for this effect in the debriefing questionnaire, with participants reporting that they were aware that they sometimes searched more than they should because they had already searched on this trial (Figure 4Bii).

Over-perseverance in later searches enabled us to examine whether people take their own future behavioral tendencies into account: if they are aware that they will later be biased in certain situations, then an adaptive strategy would be to avoid these situations. The cost of over-perseverance increases when prospective value collapses rapidly during the sequence and so we examined whether participants avoided initiating the series of searches in such trials and just took the initial offer instead. We tested this by including a regressor indexing how much prospective value might change, on average, per future search (Avg.ProspValueChange, see Figure 3B, bGLM5a). Indeed, participants searched less as a function of this factor (Figure 3D, t_24_ = −5.03; p < 0.001). Essentially, participants pre-emptively avoided sequences requiring extensive future recomputation of prospective value. This is sensible because performance in such sequences would be especially impaired by over-perseverance. We again found further support for this pre-emptive avoidance in the debrief questionnaire: some participants reported that they pre-emptively avoided trials for fear of over-persevering (Figure 4C). Inclusion of the debrief questionnaire in the online sample allowed us to examine more directly whether the pre-emptive avoidance was a response to awareness of the over-perseverance bias: first, we found that, across participants, those who were more aware that they over-persevered also reported using more pre-emptive avoidance (Figure 4D, non-parametric correlation: Kendall’s tau = 0.27, p = 0.018, n = 51). Second, we found that the subjective awareness of a bias to over-persevere moderated the relationship between the behavioral measures of the over-perseverance bias and the pre-emptive avoidance (Figure 4Ei, t_43_ = −2.14, p = 0.038, n = 47). As is illustrated in Figure 4Eii–iii, this moderation effect was driven by the fact that only those participants who reported awareness of an over-perseverance bias showed pre-emptive avoidance (t_43_ = −2.63, p = 0.012).

### Value Signals at the Initial Decision and Decision Sequence Planning

**Figure 5:**
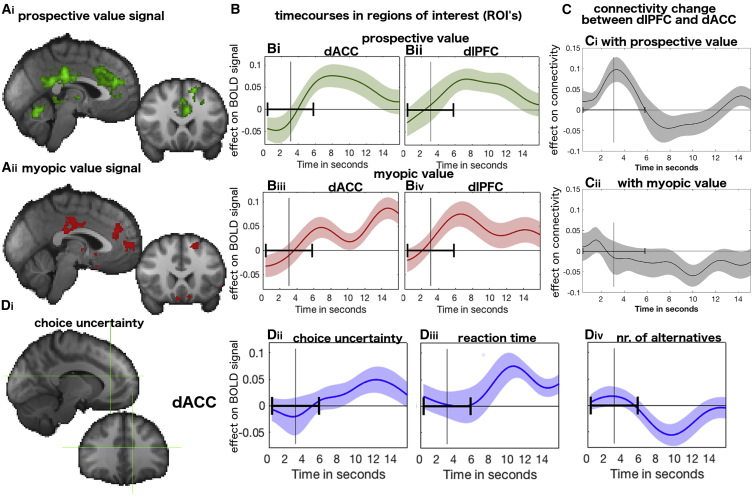
Main Neural Decision Signals (A) Whole-brain effects of prospective value (green, Ai) and myopic value (red, Aii) when the initial search decision is made. Parts of dACC and dlPFC and posterior cingulate cortex are sensitive to both aspects of value (p < 0.001, cluster-corrected; see also Table 1). (B) Time course of the regression weights for prospective (green) and myopic (red) value on brain activity in dACC and dlPFC. (C) Connectivity between dACC and dlPFC is increased when prospective value is high as opposed to low (Ci) (see Figure S6A for regression instead of split). Although both dACC and dlPFC are also activated by myopic value, this form of value does not lead to the same connectivity effects; there is no difference between dACC-dlPFC connectivity when myopic value is high or low (Cii). (D) Value-related activity in dACC is not due to difficulty. A whole-brain analysis time-locked to decision onset and using the same statistical significance thresholds as used in analyses of value-related signals found no evidence that a choice uncertainty-based metric of difficulty activated dACC at the time of decision (Di). Subsequent dACC time course analyses (ROI based on the peak of prospective value effect shown in crosshair [x = −10, y = 34, z = 18], but using anatomical ROI of rostral cingulate zone yields very similar results) found activity at the end of the trial reflected choice uncertainty (Dii) and reaction time (Diii); dACC activity was only protracted when it was difficult to select a response and reaction times were long. (Div) Although the number of alternatives might intuitively be thought to make a trial difficult, they were not associated with an increase in dACC activity (whole-brain result in Figure S5C). Time courses show means and standard error of the mean (shaded area). Vertical black line in all plots illustrates average reaction time across all participants. Horizontal ranges (in black) show the mean SD around the mean median reaction time across participants.

We used whole-brain fMRI analyses to explore the neural basis of prospective planning and initiation of decision sequences. Most notably, we found that activity in the dorsal anterior cingulate cortex (dACC, z = −4, y = 22, z = 26, Table 1), dorsolateral prefrontal cortex (dlPFC, x = −24, y = 36, z = 42, Table 1), and posterior cingulate cortex (PCC, x = −6, y = −48, z = 22, Table 1) was sensitive to both prospective and myopic value (Figures 5A and 5B). A formal conjunction analysis confirmed that both signals were present in partly overlapping voxels in each of these regions (Figure S5A; Table S2).

**Table 1:** Activation Table of Brain Analyses

	x	y	z	Max *Z* Score	p Value	No. of Voxels
fGLM1						

**Prospective value (activation)**						

Posterior cingulate cortex	−6	−48	22	4.06	2.97E-18	2,078
Pre-SMA, 8 m/9 (left)^a^	−16	20	54	3.98	9.30E-14	1,402
Pre-SMA, 8 m/9 (left)^a^: Dorsolateral prefrontal cortex	−24	36	42	3.67	–	–
Pre-SMA, 8 m/9 (left)^a^: Rostral cingulate cortex area 24^c^	−10	34	18	3.94	–	–
Thalamus (left), extending to hippocampus	−14	−16	2	3.74	1.89E-05	417
Cerebellum	0	−62	20	4.16	5.13E-05	377
Thalamus/hippocampus (right)	18	−36	0	4.1	1.61E-02	175

**Myopic value (activation)**						

Inferior parietal lobule (IPL_C, IPL_D, IPL_B)^b^	−50	−50	34	4.22	1.73E-14	1,577
Ventral striatum (extends bilaterally)	8	10	−8	4.57	3.04E-11	1,114
Pre-SMA, 8 m/9 (left)^a^	−10	8	60	3.7	8.34E-07	574
Frontal operculum (left)	−48	14	−4	4.08	4.29E-05	401
Posterior cingulate cortex (area 23)^c^	−4	−28	30	3.69	1.85E-04	342
Dorsal anterior cingulate cortex (area 32d)^c^	2	38	26	3.77	2.35E-03	247
Medial frontal pole (bilateral)^c^	−2	56	8	3.52	3.41E-03	234
Middle temporal gyrus (right)	54	−18	12	3.68	3.35E-02	159
fGLM2						

**Prospective value (activation)**						

Cerebellum	0	−62	20	3.99	9.24E-06	475
Dorsal anterior cingulate cortex (anterior rostral cingulate zone, area 24)^c^	−4	20	30	3.63	1.52E-02	187

**Average prospective value change (activation)**						

Temporoparietal junction (left)^d^	−56	−50	38	4.31	7.18E-09	886
Pre-SMA/ area 8 m/9 (left-middle)	−2	26	54	3.96	1.19E-07	737
Temporoparietal junction_p/a (right)^d^	58	−48	32	4.02	2.30E-05	465
Middle temporal gyrus (left)	−64	−34	12	3.59	4.59E-02	161

**Decision phase (neural) × prospective value (behavior)**						

Perigenual ACC (area 32pl, left)^c^	−16	50	4	4.15	3.76E-10	1,496
Lingual gyrus/percuneous (right)	14	−54	4	4.83	1.81E-03	380
Perigenual ACC (area 32pl)^c^	−4	46	0	4.21	6.55E-03	311
Primary motor cortex (left)^c^	−16	−28	70	4.06	4.12E-02	220
White matter (right), extending to hippocampus	40	−32	−6	4.17	4.59E-02	215

**Average prospective value change (neural) × average prospective value change (behavioral)**						

Perigenual ACC (area 32pl)^c^	16	42	2	3.86	8.34E-07	610
Lateral occipital cortex (left)	−46	−64	40	4.73	5.54E-04	317

Footnotes refer to the names of anatomical atlases used for labeling.

a Neubert cingulate orbitofrontal (Neubert et al., 2015)

b Mars parietal connectivity (Mars et al., 2011)

c Neubert cingulate (Neubert et al., 2014)

d Mars temporal parietal junction (TPJ) connectivity (Mars et al., 2012)

The dACC has already been linked to myopic value (Kolling et al., 2012), but a number of findings indicated a central role of dACC-dlPFC interactions in relation to prospective as opposed to myopic value. This was demonstrated by conducting two psycho-physiological interaction (PPI) analyses to investigate the relationship between activity in the two areas as a function of either myopic value or prospective value. We compared the impact of dACC activity on dlPFC (in a regression, controlling for all other task variables) after median-splitting the trials, first, as a function of prospective value, then as a function of myopic value (Figure 5C). Using a leave-one-subject-out procedure to avoid biased tests of positive or negative peaks, we found a significant increase in connectivity as a function of prospective (t_23_ = 3.26, p = 0.003) but not of myopic value (t_23_ = −2.05, p = 0.052); in fact, if anything, myopic value led to a late decrease in connectivity.

Note that the increase in prospective-value-based connectivity between dACC and dlPFC occurred before the time when the prospective value signal peaked in each of the two areas. Further analysis revealed that prospective-value-based connectivity peaked at the same time that the prospective value signal was rising fastest in each area (the temporal derivative of prospective value was highest: Figure S6B). In other words, prospective-value-based connectivity is coincident with the genesis of the prospective value signal in dACC and dlPFC.

### Prospective Value Signals Are Not Confounded by Difficulty

There has been controversy over whether myopic value can be dissociated from the difficulty associated with a decision (Kolling et al., 2016a, Kolling et al., 2016b). The type of difficulty that has been proposed to drive dACC (also called “choice uncertainty”) is related to the difference, in inverse, of the absolute value between two choices—in other words, the more similar the two options, the harder the choice. While in a previous experiment (Kolling et al., 2012) there was a positive correlation between myopic value and decision difficulty, here difficulty was negatively correlated with both myopic (r = −0.270) and prospective value (r = –0.306). Therefore, if it is a confound with difficulty that really drives dACC activity, then both myopic and prospective value should negatively affect dACC. However, both myopic and prospective value are associated with an increase in dACC activity (Figure 5A). Moreover, all our analyses, such as in Figure 5A, are from a GLM controlling for difficulty. We also did not find cluster-corrected difficulty-related activity on the whole-brain level (Figure 5Di). A more thorough look into the time course of dACC activity reveals that this was because difficulty only had an effect on dACC very late in the trial (Figure 5D), approximately 6 s after a decision could be made and the effects of prospective and myopic value became apparent. One way to interpret this is that myopic and prospective value are initially driving dACC activity for evaluative purposes. However, when decisions are harder, participants take longer, and therefore dACC activity also persists for longer.

**Figure 6:**
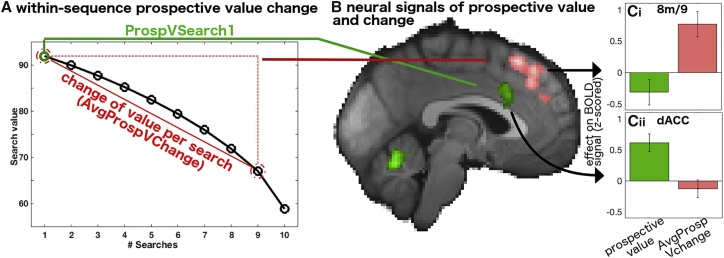
Neural Signals of Within-Sequence of Prospective Value (A) Illustration of initial prospective value and change in prospective value as the number of remaining sequences are depleted as the search horizon decreases. (B) The effect of average change in prospective value (pink) in dorsomedial frontal cortical areas 8 m/9 is shown together with the main effect of prospective value (green) in the same analysis (fGLM2). The prospective value effect is more circumscribed in this analysis because prospective value and average change in prospective value are correlated. Nevertheless, the main effect of prospective value remains centered in the RCZa sub-region of dACC (see Figure S5B). Activity is time-locked to the initial decision period, p < 0.01, cluster-corrected. (C) Region-of-interest (ROI) analyses of the BOLD data extracted from area 8 m/9 (ci) and dACC (cii), showing the effect of prospective value per se (green) and average change in prospective value (pink). Note that as ROIs were selected based on activity (change in prospective value for 8 m/9 and prospective value for dACC), statistical tests were only performed for the other regressor, respectively, showing that prospective value per se did not activate 8 m/9 (p > 0.1) and that the average change in prospective value did not affect activity in dACC (p > 0.6).

We also examined the impact of three other possible indices of difficulty that have less frequently been used but also conform to intuitions of difficulty. First, reaction time was again only associated with late dACC activity (Figure 5Diii). This is further evidence that residual variance in dACC activity at the end of the trial reflects difficulty. Second, we looked at the effect of number of search alternatives; more alternatives might make decisions harder. However, for this factor dACC activity was decreased rather than increased (Figures 5Div and S7C) (it had no direct effect on choices but affected reaction times). Finally, we also considered a very different possibility of a very high level form of difficulty; specifically, we looked at expected mental effort associated with a decision sequence. This was captured by the average change of prospective value expected during the sequence; the more prospective value changes during the sequence of decisions, the more the decision rule needs to be revised. This measure is unassociated with dACC activity (Figure 6C). This is discussed in the next section.

### Medial Prefrontal Neural Signals of Change in Prospective Value

In addition to prospective value, we were also interested in neural signals that might allow participants to anticipate future adjustments in prospective value. For this, we used an additional regressor of how much prospective value might change on average for each decision in the sequence (Figure 6A). The fMRI analysis was thus like the behavioral analysis showing initial decisions to begin a search sequence were promoted by search value but were deterred when the change in prospective value during the sequence was likely to be high (Figure 3D). Both regressors were correlated because the amount of prospective value determines how much it can fall during the course of searches (prospective value and average change in prospective value correspond to starting point and slope in Figure 6A (r = 0.78; see Figure S7D). Due to this correlation, there is now a somewhat smaller, but nevertheless significant and robust prospective value signal in dACC (x = −4, y = 20, z = 30, Table 1; Figure 6B). To confirm the effect is indeed present and unchanged in an anatomically defined area within dACC, the anterior rostral cingulate zone (RCZa as defined by Neubert et al., 2015), we show the overlap between the RCZa anatomical mask and the prospective value effect estimated in both GLMs (Figure S4B). In addition, the analysis also revealed a strong effect of prospective value change in a medial frontal region (Figure 6B) outside cingulate cortex. The activation location now straddled medial areas 8 (8 m) and 9 just anterior to and possibly extending into the pre-supplementary motor area (pre-SMA) (x = −2, y = 26, z = 54, Table 1). Thus, while dACC signals the prospective value estimate that leads participants to begin a sequence of searches, the adjacent area 8 m/9 region signals an important feature of the upcoming sequence: the rate at which the prospective value estimate will change. Region-of-interest (ROI)-based analyses showed that in fact each of the two regions did not possess the signal present in the other region (Figure 6C, dACC: change in prospective value; t_23_ = −0.90, p = 0.379; 8 m/9: prospective value; t_23_ = −1.56, p = 0.13).

### Neural Signals in Perigenual ACC Correlate with Variation in Prospection

**Figure 7:**
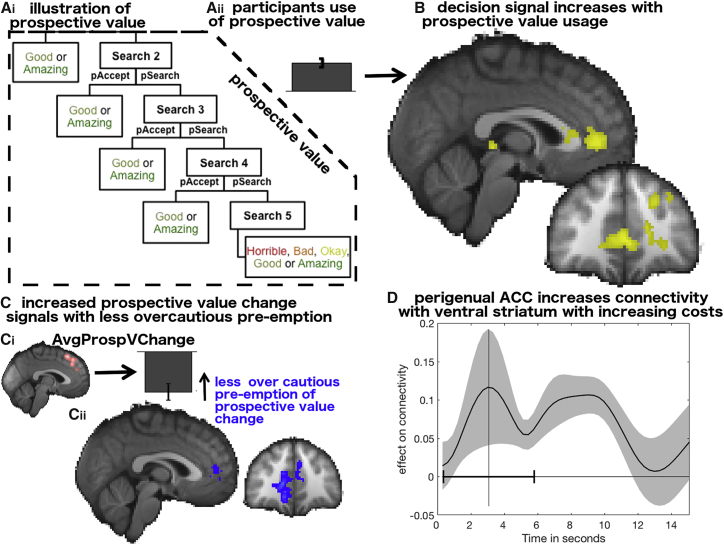
Neural Effects of Individual Differences in Prospection (A) Illustration of prospective value in the tree-search model (Ai); participants’ use of the prospective value as given by the logistic regression shown in Figure 3A (Aii). (B) Correlation of the amount of prospective value usage as indexed by the regression with the neural main effect at the decision stage. At the time of decision-making pgACC is more active in participants that are more prospective (p < 0.01, cluster-corrected). (C) The average change in prospective value, which requires adaptation of search strategy, leads to activity in areas 8 m/9 (pink) and also to avoidance of initially engaging in such decision sequences (Ci, behavioral regression weight shown in Figure 3D). However, variation in pgACC activity related to the same regressor (Cii, in blue) is predictive of less over cautious pre-emptive avoidance of such search sequences (p < 0.001, cluster-corrected). (D) Connectivity between pgACC and the ventral striatum increased significantly as a function of search costs; when there was an increased need to overcome costs, activity in ventral striatum and pgACCs is more strongly linked. See Figure S7 for ROI analyses showing the effects of costs (and other regressors) in pgACC and ventral striatum. Time course shows means with the shaded area being the SEM. Vertical line show average reaction time and horizontal line shows reaction time range.

As our task had many different dimensions to consider and it was up to the individual participants how much to weigh each constituent aspect of value when making a decision, we wanted to test whether any specific brain regions were more active when the initial decision was more prospective. We found that perigenual ACC (pgACC) was among the regions in which there was more activity during decision making (decision phase main effect) in participants who used prospective value more to guide their behavior (Figure 7B, x = −4, y = 46, z = 0, Table 1). In other words, overall decision activity in pgACC is predictive of the degree to which prospective value will drive participants’ behavior. Intriguingly, pgACC activity does not carry a strong parametric prospective value signal (Figure S7). Nevertheless, our result shows variation in pgACC’s decision-related activity is associated with variations of the impact of prospective value, which itself is encoded in dACC. The importance of pgACC in decision making is being increasingly recognized, despite the weakness of parametric value signals. Accordingly, it has also been highlighted in studies of individual differences in activity in this region (Gusnard et al., 2003, Kolling et al., 2012).

One feature of search sequences deterring participants from searching was the degree to which within-sequence adaptation was likely to be required. Using our neural data, we wanted to know whether there is activity, which might oppose such avoidance. Specifically, we looked for increased signals related to the average change in prospective value in participants less likely to be deterred by such changes during initial planning. Again, we identified pgACC (Figure 7Cii, x = 16, y = 42, z = 2, Table 1).

### Perigenual ACC Interacts with Ventral Striatum to Overcome Costs

So far, we have seen that high pgACC activity levels predicted both that a participant would be influenced by prospective value (Figure 7B) and that they would be undeterred by the likely need to readjust prospective value estimates (Figure 7C). However, this still leaves open the question of what aspect of search behavior is enabled by pgACC. Previous studies, mainly conducted in rodents (Croxson et al., 2014, Friedman et al., 2015, Rudebeck et al., 2006) but in one case in humans (Kolling et al., 2012), have suggested that pgACCs, or areas sharing homology with pgACC, are important when the costs of taking a course of action have to be considered but overcome. Intriguingly, these studies have suggested that pgACC or its homolog might not encode the costs in a simple way. Instead, changes in its interactions with the striatal regions, such as striosomes, that predominate in ventral striatum may underlie preparedness to pay a cost in order to achieve a reward goal.

We therefore conducted connectivity analysis between bilateral pgACC and ventral striatal ROIs. Both regions were anatomically defined (see STAR Methods). We used a PPI approach as above. We estimated pgACC-ventral striatal connectivity in trials involving costs as opposed to no costs. We found that, indeed, pgACC-striatal connectivity increased with increasing costs (Figure 6C, t_23_ = 2.76, p = 0.011). Intriguingly, however, there was no evidence that parametric changes in either pgACC or ventral striatum covaried with parametic changes in costs (Figure S7).

## Discussion

Sequential decision tasks are ubiquitous in natural environments when animals forage (Kolling and Akam, 2017, Stephens and Krebs, 1986), but they are also part of contemporary human life (Pearson et al., 2014). This relationship to everyday decisions could make it particularly useful for the understanding of psychiatric disorders (Addicott et al., 2017, Scholl and Klein-Flügge, 2017). This study demonstrates humans making sequential decisions are not only considering their immediately available options. Instead, they incorporate the average value of future options, their variability, the time frame or search horizon, as well as search costs. Furthermore, they even consider their own capacities and preferences for making decisions in the future.

### A Model for Prospective Sequential Decision Making in Known Environments

We have previously shown that in complex multi-alternative decision-making scenarios people consider both the value of the current or default choice with which they are engaged but also the average value and best value of alternative opportunities (Boorman et al., 2013, Kolling et al., 2012, Kolling et al., 2016b, Scholl et al., 2015). The average value of alternative options resembles what we termed myopic value in the current study. As previously, the immediate or myopic value of alternative options influenced decision making in the present study (Figure 3A). In addition, however, we showed that, when people are given the opportunity to explore alternatives over an extended time horizon, their decisions are guided by an estimate of another quantity that we call prospective value (Figures 1C and 3A). This prospective value was derived from a decision-tree model, i.e., a model of participants imagining what could happen as a result of their decision rules in an environment and how they might respond to new opportunities arising (e.g., “I will only accept encountered offers that are at least 50 points worth. Otherwise, I will keep searching for better alternatives. How good is this strategy?”).

While participants’ behavior can only be explained by assuming they use prospective values and not simply heuristics based on myopic value information, this does not necessarily mean that participants performed a full decision-tree search on each trial, i.e., that they imagined every possible decision rule they could use and every possible scenario that could arise based on applying this rule. Instead, they may have used approximations of the optimal prospective strategy. One possible short cut is to chunk very similar alternatives together. This would reduce the amount of computation needed as fewer possible thresholds would be considered. Additionally, participants’ behavior on later searches within a sequence revealed one approximation of prospective value, namely, that they assumed exponential decay of the initial prospective value across decisions in a sequence. Equally participants might use a complementary approach when they initially estimate prospective value: they might estimate prospective value for the situation in which one or two searches only are available and then extrapolate “backward” to approximate the prospective value of searching when the available numbers of searches is higher. In short, different simplifications that participants could use to compute prospective value are possible. Importantly, however, these simplifications all require some form of prospective value estimation and that in some way participants plan or imagine their future behavior.

Although the task design obviated any need for learning, we note that when agents are still learning about their environments they should also favor exploration of more variable environments when there is a long time horizon (Wilson et al., 2014, Zajkowski et al., 2017). In the real world, both factors (directed exploration and prospective value) could work together to further drive a preference for variability, novelty, or surprise in resource rich or safe contexts.

Another finding that underscored participants’ prospective planning when making their initial decisions was their consideration of their own biases. As participants progressed through an environment, they generally adapted their behavior appropriately (as search value decreased with depleted searches). However, we also found that participants were biased to over-persevere, i.e., to reject offers and keep looking for alternatives, and they did so more if they had already rejected many offers (Figure 3C). Importantly, we found that participants tried to counter-act this over-perseverance bias by pre-emptively avoiding environments with extensively changing search values during the sequence of decisions (Figure 3D). Participants’ subjective reports revealed that some were explicitly aware of these biases (Figure 4Bii) and that such awareness drove a direct relationship between over-perseverance biases and pre-emptive avoidance (Figure 4E).

### A Neural Circuit for Prospective Decision Making

We have previously found that the myopic average value of alternatives and value of the best alternative are reflected in the activity of dACC (Boorman et al., 2013, Kolling et al., 2012, Kolling et al., 2016b, Scholl et al., 2015, Scholl et al., 2017b), and again this was true in the present study (Figure 5Aii). It has been suggested that such reports are due to a confound between myopic value and choice uncertainty. In the current study, however, we were able to rule out this possibility as well as the possibility that three alternative indices of difficulty were the cause of the myopic value signal in dACC (Figures 5D and 6C). Specifically, both myopic and prospective value signals in dACC were time-locked to the onset of the decision independently from choice uncertainty and reaction time effects that only emerged late in a trial. In summary, the results suggest that dACC activity reflects myopic and prospective values, both key decision variables, but, when decisions are difficult and take longer, then dACC is also active for longer.

In addition, however, in the present study we were able to show that activity in dACC also reflects prospective value (Figure 5Ai). Again prospective value was dissociable from any metric of difficulty (Figures 5D and 6C). Moreover, prospective value, unlike myopic value, was an important determinant of the strength of interactions between dACC and dlPFC. These interactions occurred at an early point in the decision process and peaked when the increase in prospective value (temporal derivative of prospective value) peaked (Figure S6B). The interactions between dACC and dlPFC that we identified were revealed by correlations in blood-oxygen-level-dependent (BOLD) signal levels but probably correspond to interactions between the same areas established in neurophysiological studies (Khamassi et al., 2015, Oemisch et al., 2015, Voloh et al., 2015). These experiments demonstrated changes in dACC-dlPFC following reward or non-reward feedback after choices were made. The current results suggest that a critical feature of such feedback is that it informs macaques about the choices likely to lead to reward in the future. This enables them to form a prospective plan for future behavior and the outcomes that might be expected from such behavior. The current results also highlight the importance of examining not just feedback activity in dACC (Fischer and Ullsperger, 2013, Ullsperger et al., 2014) but activity related to the planning of extended sequences of behavior (Procyk et al., 2000) and changing between such behaviors (Barack et al., 2017, Pearson et al., 2014). Activity in dACC reflects progress through a sequence (Juechems et al., 2017, Kolling et al., 2014, Wittmann et al., 2016) and so dACC activation for prospective value in the current experiment may reflect simulation of possible routes through decision-tree branches.

Participants’ decisions also reflected an estimate of the change in prospective value that would occur as the search horizon diminished, not just the initially estimated prospective value. Activity related to this factor was found in medial frontal area 8 m/9 (Figure 6) but was not found in adjacent dACC. Activity in nearby regions has also been reported in other reward-guided decision-making tasks in tandem with dACC activity (Kolling et al., 2014). This suggests that computation of the expected change in search value is distinct from the initial computation of prospective value (see Figure 6C). In fact, as the change in prospective value is always a reduction in this study, we coded the regressor denoting expected change in prospective value as positive (more reduction, higher regressor, but leading to reduced search value). Thus, the activity patterns in 8 m/9and dACC are profoundly different: increasing the initial prospective value makes dACC more active, whereas 8 m/9 is more active when this value will collapse faster. Thus, it was possible to make a clear distinction between variables related to initial planning and later adaptation of behavior. This is especially interesting given the broader debate about the respective function of these adjacent—and frequently co-activating—brain regions during tasks of behavioral flexibility and adaptation.

### A Neural Circuit for Motivating an Extended Sequence of Behavior

Participants were dissuaded from beginning behavior sequences in which larger changes in prospective value occurred as the search horizon diminished (Figure 3D). While such biases are potentially the result of effortful computations and planning performed in 8 m/9, the biases were reduced in participants with greater pgACC activity (Figure 7C). PgACC, partly through its interactions with ventral striatum, is important for motivating effortful sequential search decisions over time (Figures 7B and 7D). In contrast to dACC, which carried strong signals related to the planning of an extended behavioral sequence, pgACC activity was more closely related to the motivation to engage in the sequence.

Experiments with both humans and rats, in tandem with the present results, suggest pgACC exerts its influence on behavioral motivation via interactions with striosomes, which are prominent in the ventral striatum, through a well-described anatomical circuit (Friedman et al., 2015, Kolling et al., 2012). In the current experiment, participants paid a cost to make each search decision. The cost also dissuaded participants from engaging in a search sequence (Figure 3A); however, when there was an increased need to overcome costs, activity in ventral striatum and pgACC was more strongly coupled (Figure 7D).

These results suggest pgACC plays a prominent role in motivating choices, yet it is relatively little discussed in the context of motivation and decision making in human fMRI experiments. This may be because, unlike dACC, pgACC activity does not vary significantly with prospective value. Nevertheless, it is active each time a decision is taken and its importance is underlined by examination of individual differences in decision making (Figures 6B and 6C; Gusnard et al., 2003, Kolling et al., 2012). The pgACC is distinct from more ventral granular prefrontal regions such as areas 14 and 10 concerned with other aspects of reward-guided decision making (Neubert et al., 2015).

## STAR★Methods

### Key Resources Table

**Table:** 

REAGENT or RESOURCE	SOURCE	IDENTIFIER
**Software and Algorithms**		

JATOS	Lange et al., 2015	N/A
FSL	Smith et al., 2004	N/A
Matlab	Mathworks	N/A
Presentation	Neurobehavioral systems	N/A
JavaScript	N/A	N/A
HTML	N/A	N/A
CSS	N/A	N/A
jQuery	jQuery Foundation	N/A
surveyJS	Devsoft Baltic OU	N/A

### Contact for Reagent and Resource Sharing

Please write to Lead Contact Nils Kolling at nils.kolling@psy.ox.ac.uk with requests and inquiries.

### Method Details

#### Participants

25 participants (11 female), aged 21-36 years, completed the task inside the FMRI scanner. All participants were paid £10 plus a performance-dependent bonus of between £5-11. Ethical approval was given by the Oxford University Central University Research Ethics Committee (CUREC) (Ref-Number: MSD-IDREC-C1-2013-095). One person was excluded from all neural analyses because a break in the middle of scanning resulted in difficulties in concatenating the multiple fMRI scans for pre-processing and analysis. Behavioral data from all participants was included in all analyses. Additionally, 51 participants (32 female), aged 21 to 40 years, completed an online version of the task, collected via Prolific.ac. The experiment was coded in JavaScript and the study was run using JATOS (Lange et al., 2015), using surveyJS (Devsoft Balitc OU) for questionnaire collection. Participants were paid £6.25 plus a performance dependent bonus between £0-2. Ethical approval was given by the Oxford University CUREC (Ref-number: R54722/RE001). No participants were excluded from all analyses, but some participants were excluded from some analyses if their data did not permit analysis, e.g., if for regression analyses of just the first decision on each trial, participants did not show enough variation in their behavior to fit the regression models—or if their behavioral measure was an outlier (cut-off was defined as mean ± 3 standard deviations). The number of participants included is provided for each analysis separately.

#### Training

For the group of FMRI participants: before the main task began, participants were given written instructions and trained on 20 trials with the experimenter in the room. They were allowed to ask questions. As soon as they understood the task they began the main part of the experiment consisting of 160 trials. For the online participants: training was automated so that participants read through instructions, followed by 5 training trials. They then completed a multiple-choice test, assessing task understanding. If they did not respond correctly to all questions, the training was repeated. 42 participants passed the multiple choice test on the first attempt and 9 participants on the second attempt. Participants then completed 200 trials of the main task.

#### Experimental Task

Participants had to make decisions between choice options represented by visual tokens shown on a computer monitor that were associated with different values of monetary payment to be paid at the end of the experiment. On each trial, participants were shown one option that we refer to as the “offer.” Its value was defined by the position of the dial of a clock-like stimulus (two different colors were used to indicate two different currencies, one being twice as valuable as the other; value range = 5 to 280 points). We used dials to indicate value; we wanted to give participants an intuitive sense of value without showing numbers explicitly. We used two colors to represent different currencies to further increase the range of option values. This allowed us to show very large but very rare outcome values. The presence of such options on some trials is important because participants pursue such options when many searches were available. The offer was the default option that the participants would have unless they decided to search for a better alternative instead. They were told that if they decided to search a new offer would be randomly drawn from the alternatives with replacement. There were between two and six alternatives in each trial.

Specifically, if participants decided to engage and stay with an offer, and pressed the corresponding button, they would proceed to the next stage of the trial. If, however, they pressed the other button and thus decided to search, one out of the alternatives was presented to them as their new offer. The probability of each alternative being drawn was equal to the proportion of the overall area for alternatives occupied by that particular alternative (Figure 2Ai). In other words, alternatives occupying large areas were more likely to be drawn than the ones occupying small areas.

Every time the participants opted to search they incurred the fixed cost within a given trial. However, across trials the costs varied across three levels, none (zero points), low (six points) and high (twelve points). The costs were indicated to participants by “Costs: none,” “Costs: low” or “Costs: high” being shown on the computer monitor.

Participants could search only a maximum number of times indicated by a number next to the alternatives. The maximum number of searched varied between trials (initial values were between one and ten searches).

At the decision stage the alternatives and the search horizon were presented together and the costs and current offer were presented separately. The order in which the information was presented was randomized across trials. Each piece of information was presented for a certain duration before the next piece was also revealed, with the duration between presentations drawn randomly from a Poisson distribution that depended on the feature revealed. The mean duration for the presentation of information about the alternatives and number of searches was 4.2 s (range 3.8–4.8 s, standard deviation [SD] 0.2 s). For information about the costs and current offer the duration was 1.2 s (range 0.8–1.7 s, SD 0.2 s). Only after all three presentation stages could a choice be made when a question mark appeared on the screen after on average 2.8 s (range 0.2–13 s).

After the first decision, if the offer was selected, the frame surrounding the offer would turn yellow and the frames around the alternatives turned red to indicate the choice the participant had taken. It remained this way for, on average, 4 s (SD = 1.8 s). If however, search was chosen then, on average, after 4 s (SD = 1.8 s), the new offer was revealed and subsequently a series of further search decisions could be made at 200 ms intervals until all the opportunities for searching had run out or the participant had decided to keep an offer.

Regardless of whether searches had been made or not, in the last stage of the trial, the offer currently held was shown alone in the middle of the screen for a further 3.9 s (range 2–7.2 s, SD 1.3 s) after which the outcome was presented (win or lose) for another 1.5 s (range 1.1–2.1 s, SD 0.2 s). There was always a 50% chance of winning in this stage that was independent of the participants’ previous choices. Participants were instructed about this and furthermore to ensure its credibility, all past wins and losses were counted and presented to the participants at the outcome time, to reassure them that losses and wins averaged out at about 50%. This manipulation meant that neural activity associated with the decision time was not confounded with participants having certain knowledge of reward receipt. Instead, reward receipt related activity should only occur at the outcome.

This was followed by an inter-trial-interval (ITI) until the next trial started. The ITI was, on average, 4 s (range 2–7.2 s, SD 1.3 s).

#### Task differences in the online study

As participants could only be given standardized task instructions, rather than “in person” instructions by the experimenter, the graphic layout was changed: the alternatives were represented on a “wheel of fortune” and “searching” was referred to as “spinning the wheel.” Instead of using the symbols for currencies and cost as before, we used explicit numbers. The outcome phase, with the 50% probability of getting the accepted offer, was omitted as no neural data were acquired. The timings of the task were sped up to maintain motivation and task engagement: delay before the first response ranged from 3 to 6 s (uniform distribution); delay between decisions to search and new offer: 0.3–2 s (uniform distribution); delay to next trial: 1.5 s (fixed). To ensure that despite these differences in task design, behavior was nevertheless comparable, all analyses from the main manuscript were replicated (Figure S3).

#### Questionnaires

In the online sample only, participants completed an extensive debriefing questionnaire to gain additional insight about their behavioral strategies after the behavioral task. The questions reported in the main manuscript were: Q8) “How much did you take the likelihood of getting your preferred outcome eventually (even if not on the first spin) into account?” (answers in 7 numerical steps from “not at all” to “very much”); Q9) “In the beginning of each round, did you feel like you only thought about the very first spin or did you plan further ahead?” (possible answers: “no planning”, “very first spin”, “2-3 spins”, “4 spins”, “5 or more spins”) [note that for ease of explanation, in the version of the task for the online participants, decisions to search were referred to as “spins” of a “wheel of fortune”]; Q10) “Were there ever any offers that you banked later in a round that you would not have banked at the beginning of a round?” (answers in 7 steps from “never” to “always”) [note that in the version for the online participants, decisions to accept an offer were referred to as “bank an offer”]; Q12) “Did you ever feel like you continued spinning the wheel just because you had spun the wheel already this round, rather than because you really thought this was the right thing to do?” (answers in 7 steps from “never” to “always”) [note that in the online sample, trials were referred to as “rounds” in a game]. Q13) “Did you ever avoid starting spinning the wheel in the first place in a round because you were worried that you might end up spinning it too many times?” (answers in 7 steps from “never” to “always”). Participants also completed various psychiatric questionnaires that were not analyzed for this manuscript.

#### Computational model

The aim of the model is to compute the value of searching taking into account the possibility for future searches given the current search horizon (available number of searches), the alternatives’ properties, and search costs. This model-based value can then be compared to the current offer to make the decision of whether to accept the offer or to search for a better alternative. To find the value of searching, the model simulates a series of full decision trees, each time applying a different acceptance-threshold, i.e., a value above which it would accept a new offer and otherwise reject it to search again. Examples of decision trees are shown in Figure S1. For each end-state of the decision tree (i.e., “branch”), the model computes the probability of this state given the acceptance-threshold. It then multiplies this probability with the reward value of the end-state outcome (taking into account both the reward received and the cost of the number of searches taken to get to this point). The value of searching (VSearch) thus becomes:

$$\text{VSearch}=\sum_{\text{s}=1}^{\max\text{Search}-1}[\text{p}{\left(\text{Search}\right)}^{\text{s}-1}*\left(\text{AcceptUtility}-\text{s}*\text{cost}*\text{p}(\text{Accept}\right)]+\text{p}{\left(\text{Search}\right)}^{\max{\text{Search}}^{-1}}*\left(\text{Myopic}\;\text{utility}-\max\text{Search}*\text{cost}\right)$$

Where p(Search) is the probability of searching which is equivalent to the summed probabilities of the alternatives below a given acceptance threshold. P(Accept) is the opposite: the probability of not searching and instead accepting an alternative in the decision tree (i.e., an offer that is received). MaxSearches corresponds to the search horizon: the maximum number of searches possible in a trial and therefore the depth of the decision tree. Cost is the point loss incurred for every search decision on a given trial (Figure S1A illustrates a step-by-step derivation of the formula). Note, the model also employs a changing acceptance-thresholds at each search step; it allows for different p(Search)and p(Accept) for every step in the sequence (or level of the tree; Figure S1B). The term s corresponds to the search number within the tree (e.g., s = 1 is the first search in an environment).

#### Regression analyses

We performed regression analyses, predicting either participants’ decisions to reject an offer and search (i.e., draw a new alternative) or their reaction times. See Table S1 for a concise list of all regressions. We performed regressions on three different subsets of trials: First we included all responses throughout all trials in the analysis (bGLM1a-e). Second to examine behavior during the sequence of search decisions that occurred after the initial decision to undertake a sequence, we carried out a similar analysis but included only the responses after the initial one on each trial (bGLM2-4). Third, we carried out the complementary analysis and focused only on the initial decision whether or not they undertake a sequence; this analysis included only the very first response on each trial (bGLM5). To analyze decisions, we used a logistic regression. To analyze reaction times, we used a log link function and a gamma distribution (model comparisons suggested this combination as the best fit for our reaction time data). Regressions were performed in MATLAB (glmfit). In the reaction time analyses, we also included a separate regressor to indicate whether a search was the first one in a trial as reaction times were markedly different between the first and subsequent searches (Figures 2C and S3A). All regressors were z-score normalized. Regression analyses were performed separately for each participant. Regression weights were tested for significance at the group level using t tests.

#### Analysis of all decisions

The main aim of the analyses was to test whether participants’ behavior was influenced by prospective and myopic values (Figure 3A):

bGLM1a:

$$\log it\left(Y\right)=\beta_{0}+\beta_{1}\;OfferValue+\beta_{2}\;SearchCost+\beta_{3}\;MyopicValue+\beta_{4}\;ProspectiveValue$$

We also tested whether people really planned their future behavior or whether instead they used some simpler non-prospective heuristic. For this, we built two models in which we included different non-prospective heuristics participants could use instead of the model-derived prospective value. To test whether prospective value explained behavioral variation beyond those heuristics, we included prospective value as an additional regressor. In the first of these models, we included as non-prospective heuristics:

bGLM1e:

$$\log it\left(Y\right)=\beta_{0}+\beta_{1}OfferValue+\beta_{2}SearchCost+\beta_{3}MyopicValue+\beta_{4}HighestAvailableMagnitude+\beta_{5}MagnitudeRange$$

We then tested whether prospective value still had a significant impact on behavior when controlling for these other factors. To supplement the t test of the regression weight for prospective value, we performed a model comparison: to test whether the model fit was improved by including prospective value in addition to a simpler heuristic, we ran another regression (bGLM1e2) including all regressors from bGLM1e, and additionally the prospective value. Model fits were then compared using Akaike’s information criterion (AIC). We plot the AIC difference scores (between the two models of interest) for each participant. We also compute the total AIC difference scores, i.e., by summing across all searches done by all participants.

For the construction of a similar additional set of non-prospective heuristics (see Figure S2), we noted that for example prospective value as estimated from a decision tree is a non-linear function of search horizon and the variation, indexed by standard deviation, in the values of the alternatives (participants can keep searching for one exceptional high value alternative or reject really bad outcomes if the search horizon is long and many opportunities to search again remain). Nevertheless prospective value can be approximated by the linear effects and interactions of search horizon, standard deviation of alternative values, and costs. We therefore examined whether the model-derived estimate of prospective value explained search decisions over and above the linear effects of search horizon, standard deviation of alternative values, and costs in the following GLM:

bGLM1b:

$$\begin{array}{l} logit\left(Y\right)=\\ \beta_{0}\;+\beta_{1}OfferValue+\beta_{2}SearchCost+\beta_{3}MyopicValue+\beta_{4}ProspectiveValue+\beta_{5}AvailableSearches\;\\ +\beta_{6}Standard\;deviation\;of\;patches\;\left(STD\right)+\beta_{7}\left[AvailableSearches*EnvironmentStdev\right]\\ +\beta_{8}\left[AvailableSearches*SearchCost\right] \end{array}$$

where the variance (i.e., square of the standard deviation) of the options in the environment (standard deviation of patches, STD) was computed (for n options in an environment) as:

$$\sum_{option=1}^{option=n}\left[{\left(Magnitude_{option}-Magnitude_{average}\right)}^{2}*\mathrm{Probabilit}y_{option}\right]$$

Model fits were compared as described above.

As a third alternative strategy that participants might use instead of computing prospective value, we considered an “effortless” strategy, i.e., that participants would always reject an offer unless it was the highest, apart from on the second to last available search in a trial in which they would accept anything that was not the lowest offer:

$$\log it\left(Y\right)=\beta_{0}+\beta_{1}\text{OfferisHighestAndNotLastSearch}+\beta_{2}\text{OfferisNotLowestAndLastSearch}$$

Results of these analyses are shown in Figures 3A and S2.

Finally, we performed regressions to decide how best to include myopic value in the analyses of the neural data. Instead of including myopic value as a single regressor, we split it into two separate regressors carrying the myopic value of the three most likely alternatives and the rest (the main effect of whether there were more than three alternatives was removed through z-scoring of the second regressor for only trials with more than three alternatives, otherwise it was set to zero). We did this because in the task, the number of available alternatives was varied between 2 and 6 and we wanted to assess how this affected the use of less likely aspects of the alternatives when computing the average. In bGLM1c we thus included:

$$\log it\left(Y\right)=\beta_{0}+\beta_{1}OfferValue+\beta_{2}SearchCost+\beta_{3}Myopicvalue\left(three\;most\;likely\;alternatives\right)+\beta_{4}Prob\;Myopicvalue\left(remaining\;alternatives\right)+\beta_{5}ProspectiveValue$$

Results of this analysis are shown in Figure S4.

#### Analysis of later decisions

In these analyses we tested whether decision and reaction times in searches after the very first one in each environment.

The first aim of the analyses performed on the later decisions in each trial was to assess whether participants adjusted their search values over the course of a trial (as there were fewer and fewer searches remaining) and whether they were biased to either under or over persevere. For this we included two new regressors. The first was how many searches participants had already performed on the current trial. This should not influence their decisions to search or accept per se because all variables that should rationally influence their decisions were also included in the GLM. The second was how much the prospective value on the current search was lower than at the first search. Given that the model also included a regressor of prospective value at the first search, then participants who adjust their prospective value as it decreases with fewer searches remaining should show a negative regression weight for the difference between initial and current prospective value (value is always positive or zero). This is because the regressor should capture the updating of prospective value over the course of a trial.

bGLM3a:

$$\log it\left(Y\right)=\beta_{0}+\beta_{1}OfferValue+\beta_{2}SearchCost+\beta_{3}Myopicvalue+\beta_{4}ProspectiveValueInitialSearch+\beta_{5}\left(ProspectiveValueInitialSearch-ProspectiveValueCurrentSearch\right)+\beta_{6}NumberOfPreviousSearches$$

Finally, we were interested in whether participants only computed the prospective value at the initial search and how it might change and then, instead of recomputing full decision trees at every subsequent search, use the initial value and discount it as they progress through the search sequence. First, we note that an exponential function approximates the effect that number of searches has on the decrease in prospective value over the course of a trial (Figure 3B). Based on this, we took all prospective values for all searches shown to participants and fit an exponential curve to them, separately for different cost levels, in order to get an empirically derived discount function:

$$ProspVal_{currentSearch}=ProspVal_{initialSearch}*e^{\delta *\left(\frac{Searches_{done}}{Searches_{\max}-1}\right)}$$

Where δ are the discount factors that we determined from the data (separately for different search costs). We note that this analysis is solely based on what was shown to participants, not on the choices they made. The very strong correlations between prospective value derived in this way and the precise model-based tree-search derived prospective value are shown in Figure S2D.

To test whether this approximation of the prospective value changing within a sequence explained our data better than the precise model-based tree-search derived prospective value, we ran two regressions (bGLM4a+b) that contained as regressor of interest either the true prospective value or the exponential curve-based approximation (in both cases all other regressors were as in bGLM1a). We compared the fits of these models using AIC.

#### Analysis of initial decisions

Finally we also looked at initial decisions in each trial. We wanted to test whether participants even at the first decision in a sequence, anticipated whether the subsequent search sequence might be taxing because it required readjustment of the search value. To index this we included a regressor of interest corresponding to the average change in search value within a trial. We reasoned that participants might be less likely to begin searching if they knew that they would have to adapt their search strategy in the future. This potential change in prospective value per search was computed as:

$$Avg\mathrm{Pr}ospValueChange=\frac{ProspVal_{Search1}-ProspVal_{MaxSearches-1}}{MaxSearches}$$

We used the prospective value at the second to last search, because at the last search on each trial the prospective value is always zero and so there are no remaining searches to be prospective with. The prospective value at the first search is always higher than at later searches. Therefore, the value of AvgProspValueChange is always positive. In the regression, we predict that if people pre-emptively avoid such taxing search sequences then their regression weight for AvgProspValueChange should be negative.

bGLM5a:

$$\log it\left(Y\right)=\beta_{0}+\beta_{1}OfferValue+\beta_{2}SearchCost+\beta_{3}MyopicValue+\beta_{4}ProspectiveValue+\beta_{5}AvgProspValueChange$$

#### Validation of the regression analyses by simulation

To validate that our regression analyses were both sensitive if the effect of interest was present in our data and at the same time would not show a false positive effect if there were no effect in our data, we used simulations (Palminteri et al., 2017). This analysis step was also motivated by the fact that we noticed that some regressors showed high correlations and we therefore wanted to ensure that our analyses were nevertheless valid. In each case, we used a computational model to simulate data from 25 participants (same size as real dataset, FMRI sample) that made choices on each trial (using a standard soft-max decision rule) based on the same information as our real participants. Figures S2F–S2I illustrates details of all the simulations performed to verify the different regressions. We found that all our regression analyses were both sensitive to the presence of effects of interest but in addition they were selective; they showed no false positives.

#### Analyses of debriefing questionnaires (online sample only)

We tested whether subjectively reported awareness of a bias to over-persevere (Q12) moderated the relationship between over-perseverance (measured in the behavioral regression analysis of the later searches, bGLM3a, as regression weight for “number of previous searches”) and pre-emptive avoidance (measured in the behavioral regression analysis of the first search on each trial, bGLM5a, as regression weight for “average prospective value change”). The moderation analysis was done as a regression analysis across participants predicting pre-emptive avoidance (behavioral regression weight) based on over-perseverance (behavioral regression weight), awareness of over-perseverance (questionnaire rating) and the interaction of the two regressors (i.e., the moderator effect); all regressors were z-score normalized and to compute the interaction regressor, both regressors were z-score normalized before being multiplied together.

$$Preemptive\;avoidance_{behavioral}=\beta_{0}+\beta_{1}Overperseverance_{behavioral}+\beta_{2}Overperseverance_{questionnaires}+\beta_{3}Overperseverance_{behavioral}*Overperseverance_{questionnaires}$$

To illustrate the moderation affect, we performed a second analysis again predicting pre-emptive avoidance and including as regressors of interest separately the behavioral over-perseverance for participants who reported no awareness of over-persevering and for the remaining participants. As control regressor, we again included the questionnaire score for awareness of over-perseverance.

#### MRI data acquisition

Structural MRI and fMRI measurements were taken using a Siemens 3 T MRI scanner. For the fMRI, we used a Deichmann echo-planar imaging (EPI) sequence (Deichmann et al., 2003) [time to repeat (TR): 3000 ms; 3x3x3mm voxel size; echo time (TE): 30ms; flip angle: 87°; slice angle of 15° with local z-shimming] to minimize signal distortions in orbitofrontal brain areas. This entailed orienting the field-of-view at approximately 30° with respect to the AC-PC line. We acquired between 1252 and 1724 volumes (depending on the time needed to complete the task) of 45 slices per participant. Additionally for each participant, anatomical images were acquired with a T1- weighted MP-RAGE sequence, using a GRAPPA acceleration factor of 2 (TR: 2200 ms; TE: 4.53 ms; inversion time: 900ms; voxel size: 1x1x1 mm on a 176x192x192 grid) [same protocol as Chau et al., 2014].

We used FMRIB’s Software Library (FSL) (Smith et al., 2004) for image pre-processing and analysis. Analyses were run on a local computer using HTCondor and code from Neurodebian (Halchenko and Hanke, 2012) to share analyses across cores. Functional images acquired were first spatially smoothed (Gaussian kernel with 5mm full-width half-maximum) and temporally high-pass filtered (3 dB cut-off of 100 s). Afterward, the functional data were manually de-noised using probabilistic independent component analysis (Beckmann and Smith, 2004), identifying and regressing out obvious noise components (Kelly et al., 2010); we considered only the first 35 components of each participant which had the greatest potential to interfere with task data (total up to 550). We used the Brain Extraction Tool (BET) from FSL (Smith, 2002) on the high-resolution structural MRI images to separate brain matter from non-brain matter. The resulting images guided registration of functional images in Montreal Neurological Institute (MNI)-space using non-linear registrations as implemented in FNIRT (Jenkinson et al., 2012). The data were pre-whitened before analysis to account for temporal autocorrelations (Woolrich et al., 2001). Statistical analysis was performed at two levels. At the first level, we used an event-related general linear model (GLM) approach for each participant. On the second level, we used FMRIB’s Local Analysis of Mixed Effects (FLAME 1) (Beckmann et al., 2003) with outlier de-weighting and tested the single group average. The main effect images are all cluster-corrected results with the voxel inclusion threshold of z = 2.7 and cluster significance threshold of p = 0.05.

#### Whole-brain fMRI analyses

In the fMRI analysis we looked for neural signals related to how participants made the first decision on each trial. In fGLM1 our two regressors of interest were the prospective value and the myopic value, time-locked to the decision. However, a number of other control regressors were also employed.

First, we used three regressors to denote the occurrence of three phases within each trial: decision phase (when participants first decides to take the offer or search for a better alternative); search phase (when participants moved through a sequence of searches); outcome phase (delivery or non-delivery of the reward probabilistically). These were modeled with a stick regressor for the “decision phase” (i.e., duration of 0), a box car regressor for the “search phase” (from beginning to the end of the search period; average = 3.4 s), and finally a box car regressor for the outcome phase (from beginning to end of this period; average = 1.5 s). Second, a number of parametric regressors were used at the time of each trial phase. For the decision phase all parametric regressors were stick figures locked to the onset of the decision period and all with the same duration of zero as the decision phase regressor described above. They included: the value of the initial offer, the myopic value (separately for the 3 most likely options and the remaining very unlikely options, see Figure S4 for the argument for this and description of bGLM1c above)-, the decision cost, the natural logarithm of participants’ RTs, the prospective value, the standard deviation of the values of the options in the environment (as defined above) and the difficulty of the decision. Difficulty was defined as the negative of the absolute distance from indifference (50/50 choice prediction) between searching and staying, often referred to a choice uncertainty:

difficulty=−abs(p(search)−0.5)).

For this, the probability of searching was obtained using a standard soft-max decision rule whereby all choices each participant made were predicted based on the difference in search value and offer value, allowing also for an individual bias to search independent of search value:

$$p\left(Search\right)=\frac{e^{\beta *\left(Value_{Search}+SearchBias\right)}}{e^{\beta *\left(Value_{Accept}\right)}+e^{\beta *\left(Value_{Search}+SearchBias\right)}}$$

where β is the inverse temperature.

This two-parameter model was fitted separately to each participant’s data using MATLAB’s fminsearch. In the search phase parametric regressors had the duration of the search phase and included: the final offer that participants accepted, the myopic value (again, split into two separate regressors), the cost of searching, the prospective value, the number of searches done, and the standard deviation of the values of the options in the environment. In the outcome phase, the parametric regressor had the duration of the outcome phase and included: the prediction error (i.e., the magnitude of the reward outcome participants actually received minus the final offer value). We also included six motion regressors as derived from FSL’s motion correction algorithm as confound regressors.

In a second analysis (fGLM2), we tested for the neural correlates of potential future changes in prospective value. We therefore included the regressor AvgProspValueChange (computed as described above) in addition to all the regressors detailed for fGLM1. Correlations between regressors are shown in Figure S7D. To see whether the neural signals identified for prospective value, or future change in prospective value related to behavioral measures, we performed fGLM2b. Here, at the group level, we included for each participant their (z-scored) regression weights for prospective value and AvgProspValueChange (both from bGLM5 as described above).

#### Computing overlap between prospective and myopic activity

To quantify the amount of overlap between myopic and prospective value in dACC, dlPFC and posterior cingulate cortex, we used anatomical masks for these regions (Neubert et al., 2015, Sallet et al., 2013). Specifically for dACC, we included the rostral cingulate zone and area 32d (Neubert et al., 2015); for posterior cingulate cortex, we included area 23ab (note that its posterior boundary was determined by the posterior limit of the region investigated by Neubert and colleagues, 2015) while the dlPFC region included areas 8b and 9/46d (Sallet et al., 2013). In these masks, we calculated the number and percentage of voxels for each contrast that exceeded p < 0.05 or p < 0.01 as well as the percentage of voxels that showed significant activity related to both of the contrasts, myopic value and prospective value, independently. Analyses were done in FSL (FEAT), including the respective masks for pre-threshold masking and using uncorrected thresholds (i.e., no correction for the number of voxels in each ROI to facilitate comparison between the ROIs that were differently sized).

#### ROI time course and inter-regional activity correlation analyses

To illustrate the time course of the activity identified in the whole brain analyses and to measure connectivity between brain regions, we analyzed data in regions of interest. A detailed graphical depiction of the approach is provided by Scholl and colleagues' (Scholl et al., 2017a) Figure S6. In short, we extracted data from ROIs, which were spheres with a 3 voxel radius (6 mm^3^), identified in MNI standard space. We then up-sampled the time course data by a factor of 10 and cut the data into epochs aligned to the onset of the decision phase. All regressors as described above in the decision phase were included. Any time courses which were extracted from non-orthogonal contrasts are for illustration purposes only and no statistical tests were performed. Time courses with statistical tests, were either based on anatomical ROIs (such as RCZa) or based on orthogonal contrasts (connectivity effects). To test connectivity between brain areas, we used psycho-physical interaction (PPI) analyses. We note that as the connectivity analyses are orthogonal to the main effects used to identify the ROIs, there are no problems of biased sampling. In the first analysis we tested whether connectivity between dACC and dlPFC (which both showed activity with prospective value) varied with prospective value, but not with myopic value. For this we extracted the BOLD signal based on activity peaks in dACC and dlPFC. We created two separate regressors: we separately included dACC activity on the half of trials when prospective value was high or low (median split). As control regressors we included the same psychological regressors as described above, as well as the average time course from the whole brain. We then formed the contrast between the regression weights for the trials of high versus low prospective value, to determine when the correlation in brain signal between the two areas changed as a function of prospective value. For statistical testing to remain unbiased in peak selection or by the exact temporal profile of the PPI effect, we used a leave-one-subject-out procedure to find the peak time (within a window of 0.5 to 12 s) for all but the left out subject. For the left out subject we then noted down the regression weight at that time point. This was repeated 25 times, each time leaving out a different participant. We repeated this analysis for splitting trials according to myopic value. Very similarly, for statistical tests of the time courses of the impacts of regressors on BOLD activity (e.g., as shown in Figure S9), we also used a leave-one-out procedure. The only difference was that instead of extracting just the BOLD value for each participant at the peak time point (based on the peak of the remaining participants’ activity), we use hemodynamic convolution, as previously described (Scholl et al., 2017a). In the second PPI analysis, the psychological regressor of interest used to split the data into two categories was the cost level (either no search costs or costs) and the brain areas of interest were the bilateral perigenual ACC and the bilateral striatum, both identified anatomically. To test whether there is a connectivity effect when the cost needs to be overcome we have a later time window (4.5 to 12 s). The pgACC ROI was the prelimbic area 32 (p32) in the anatomical parcellaton of Neubert and colleagues (Neubert et al., 2015; threshold at 25%) and the nucleus accumbens ROI in the ventral striatum from the Harvard-Oxford atlas (threshold at 25%) from the FMRIB software library (FSL) (Smith et al., 2004).

## Supplemental Information

Document S1. Figures S1–S7 and Tables S1 and S2

Data S1. Prospective Value, Related to Figure 5 This file is the thresholded z-statistic image shown in Figure 5 for the contrast of prospective value

Data S2. Myopic Value, Related to Figure 5 This file is the thresholded z-statistic image shown in Figure 5 for the contrast of myopic value

Data S3. Choice Uncertainty, Related to Figure 5 This file is the thresholded z-statistic image shown in Figure 5 for the contrast of choice uncertainty

Data S4. Decision MaxProspVChange, Related to Figure 6 This file is the thresholded z-statistic image shown in Figure 6 for the contrast of prospective value change

Data S5. ProspValue Change Controlled, Related to Figure 6 This file is the thresholded z-statistic image shown in Figure 6 for the contrast of prospective value controlling for its change

Data S6. MaxProspVChange by Behavioral Regressor, Related to Figure 7 This file is the thresholded z-statistic image shown in Figure 7 for the contrast of correlation of the neural prospective value change signal with the behavioral effect of preemptive avoidance of the same value change.

Data S7. Decision Effect by Behavioral Effect of Prospective Value, Related to Figure 7 This file is the thresholded z-statistic image shown in Figure 7 for the contrast of the main effect of the decision phase correlation with the behavioral effect of prospective value.

Document S2. Article plus Supplemental Information
