# Supplementary material for: Prospection, Perseverance, and Insight in Sequential Behavior
Source: Neuron. 2018 Sep 5;99(5):1069–1082.e7. doi: 10.1016/j.neuron.2018.08.018 (PMC6127030; doi:10.1016/j.neuron.2018.08.018)
Supplement: Document S1. Figures S1–S7 and Tables S1 and S2 [file mmc1.pdf]

Neuron, Volume *99*

Supplemental Information

Prospection, Perseverance, and Insight

in Sequential Behavior

Nils Kolling, Jacqueline Scholl, Adam Chekroud, Hailey A. Trier, and Matthew F.S. Rushworth

Supplementary information

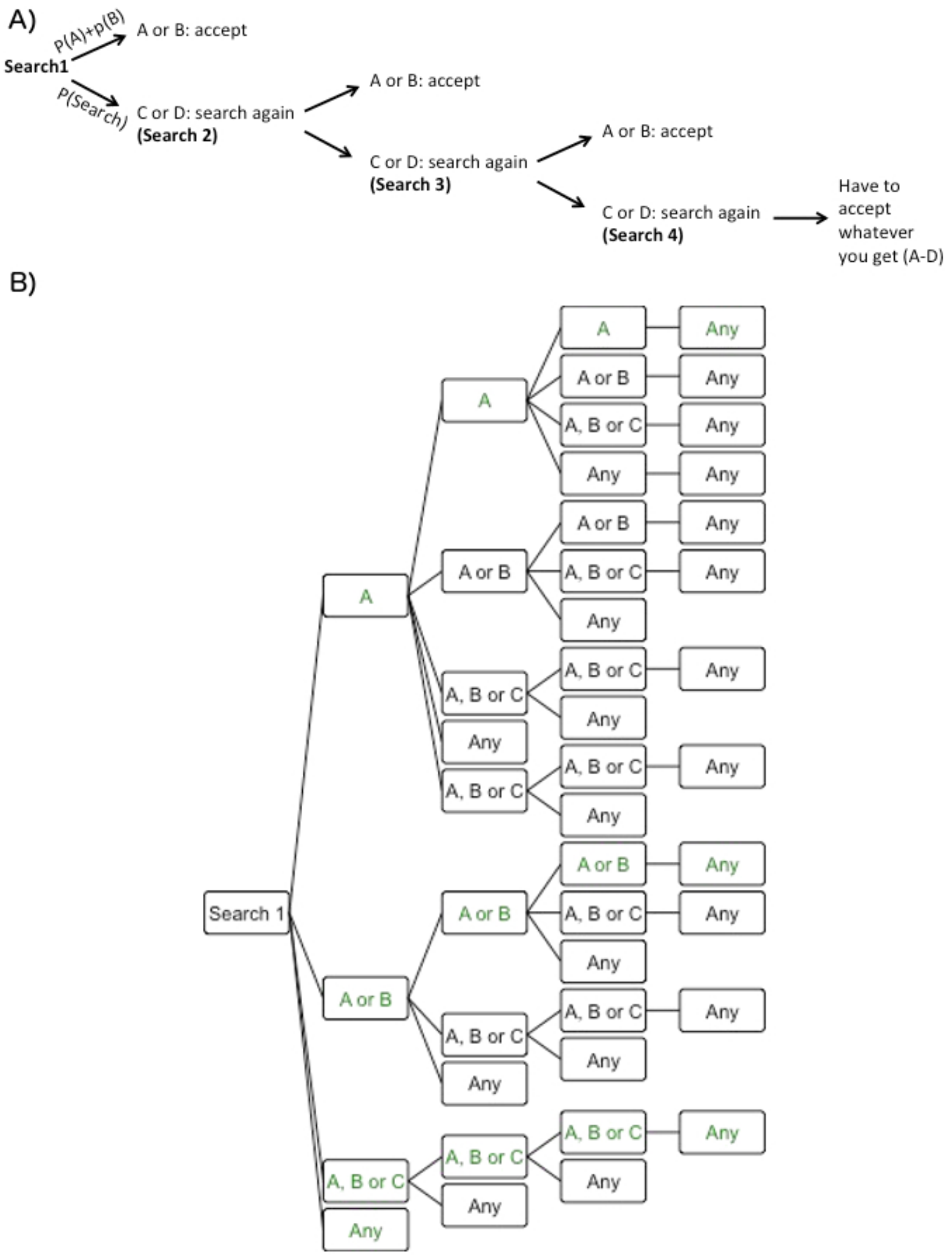

Figure S1. Decision tree to compute search value, Related to Figure 1. *A) Illustration of how the computational model computes the expected value of searching given a single example threshold. In this example there are four alternatives with magnitude A≥B≥C≥D and the number of available searches is four. The model is run for each possible acceptance-threshold (≥A (accept A only), ≥B (accept A or B), ≥C (accept A, B or C), ≥D (accept A, B, C or D). Here we illustrate the calculations if the acceptance-threshold is ≥B. The key point is that to compute the expected utility of searching at search one, the model has to compute the sum of all the branches in the decision-tree:*

$$p(A)*(mag(A)-cost) + p(B)*(mag(B)-cost)$$
$$+ p(Search)^{1}*[p(A)*(mag(A)-2*cost)+p(B)*(mag(B)-2*cost)]$$
$$+ p(Search)^{2}*[p(A)*(mag(A)-3*cost)+p(B)*(mag(B)-3*cost)]$$
$$+ p(Search)^{3}*[p(A)*(mag(A)-4*cost)+p(B)*(mag(B)-4*cost)+p(C)*(magC-4*cost)+p(D)*(magD-4*cost)]$$

with p(Search) = p(C)+p(D)
Or equally: pSearch = (1- (p(A)+p(B)), as A, B, C and D have a combined probability of 1

To simplify these equations, we can use:

p(Accept) = p(A) + p(B), and
*AcceptUtility = p(A)*mag(A) + p(B)*mag(B)*
*Myopic utility = p(A)*mag(A) + p(B)*mag(B) + p(C)*mag(C) + p(D)*mag(D)*

The search value at search step one then simplifies to:

$p(Search)^0$*(AcceptUtility – cost* p(Accept))
+ $p(Search)^1$*(AcceptUtility – 2*cost* p(Accept))
+ $p(Search)^2$*(AcceptUtility – 3*cost* p(Accept))
+ $p(Search)^3$*(Myopic utility – 4*cost)

Generalizing this, we obtain the following expression for the value of searching (as described in Methods):

$$VSearch = \sum_{s=1}^{maxSearch-1} [p(Search)^{s-1} * (AcceptUtility\ - s * cost * p(Accept)] + p(Search)^{maxSearch-1} * (Myopic\ utility\ - maxSearch * cost)$$

B) Example of full range of possible acceptance-threshold combinations for the same foraging environment as shown in A). We can re-apply the same computations as detailed in A) for all possible combinations of acceptance thresholds. This takes into account that it is possible that acceptance thresholds change over the course of a trial, in other words that a value that should be rejected when there are many searches remaining, should be accepted with fewer researches remaining. We know from human behavior (fig 3C) that people indeed decrease their estimate of the prospective value over the course of repeated searches in a trial.

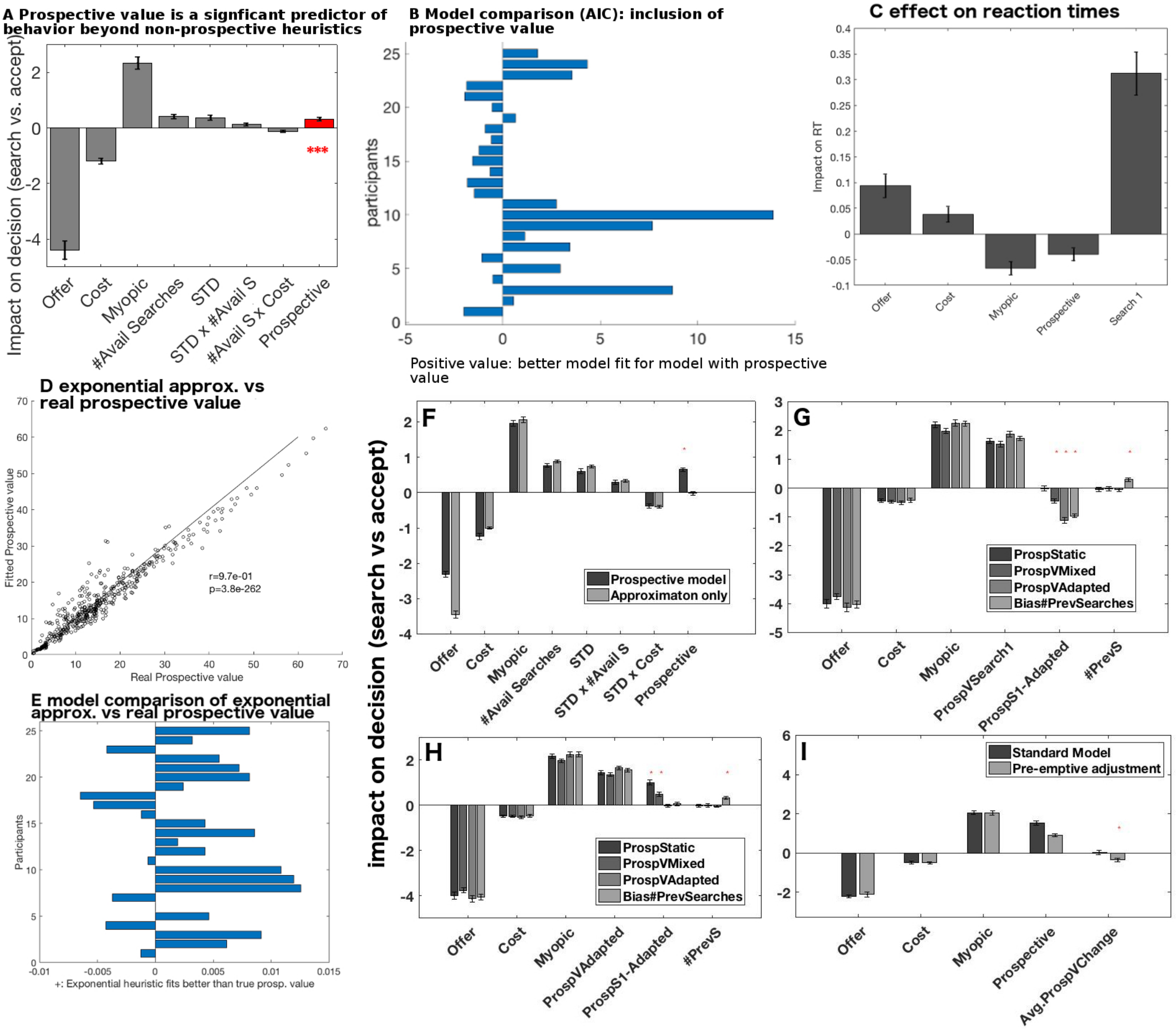

Figure S2. Dissociating prospective value from non-prospective heuristics, Related to Figure 3. *In the main text (figure 3Aii-iii), we found that a model using prospective explained participants' choices better than two alternative models using non-prospective heuristics. Here we are testing an additional model of non-prospective heuristics. These included the search horizon, i.e. the number of available searches (#Avail Searches), the standard deviation of the values of the alternative options (STD), the interaction between the standard deviation of the values of the alternative options and the number of available searches (STD x #Avail S) and finally the interaction between the number of available searches and the cost of searching (#Avail S x Cost). We included these non-prospective heuristic regressors in a regression analysis to predict search decisions as opposed to accept decisions (bGLM1b). A) We orthogonalized the prospective value with respect to all other regressors in the GLM it (i.e. we removed any variance from prospective value that was shared with the other regressors) to test whether the model-derived prospective value provided additional explanatory power even after considering the influence of the simpler heuristics. We found prospective value remained a highly significant predictor of behavior (red bar on far right; t_{24}=4.664; $p<0.001$). We note, that (unsurprisingly) the same result was found when prospective value was not orthogonalized before inclusion in the GLM. This control analysis provides evidence that people indeed decided whether to search or not by taking into account future decision states that might become available and that they did not exclusively rely on simple non-prospective heuristics. B) The same result can be found using model comparison, analogous to figure 3Aii, (summed AIC difference = 35.4). Note that in 12 participants the prospective value improves the fit, while in 13 it does not, suggesting heterogeneity in the degree to which participants use prospective value. C) To test statistically whether reaction times (RT) on the first search were slower than on subsequent*

*searches – as suggested by the historgram in figure 2C – we ran a regression analysis on the RTs of all searches in each trial (bGLM1a). We found that the first search on each trial ('search 1') was markedly slower than subsequent searches (t_{24}=7.40,p<0.001). This suggests that participants do not go through the time-consuming process of recomputing a full decision tree at later searches in the trial. D) As shown in the main text (figure 3B), the decrease of prospective value over the course of a trial follows an approximately exponential curve. Exploiting this feature, we approximated the prospective value on later searches as an exponentially decaying function of the initial prospective value and the number of searches done in the trial (see Methods, section on 'analysis of later decisions'). There was a very strong correlation between the true prospective value and the approximation fitted with this approach (r=0.97, p=3.8*10^{-262}). This thus suggests that participants might use a similar approximation to allow them to compute the prospective value on later searches without having to recompute a full decision-tree. E) We tested this by performing a model comparison of two regression analyses predicting RTs (bGLM4a and bGLM4b) that only differed in whether prospective value was the true prospective value from the model or the exponential approximation. We found some evidence that the exponential approximated prospective value explained the data better (summed AIC difference = 16.1, better fit in 16/25 participants). *** p<0.001;**p<0.01, *p<0.05.* ***F to H)*** *To validate that the regression analyses that we performed on the behavioral (decision) data were both sensitive to the presence of the effects of interest, and at the same time sensitive to the absence of such effects we simulated data from models that either did or did not have the effect of interest as a ground truth (see Methods, section 'Validation of regression analyses by simulation'). In each case, we used a computational model to simulate data from 25 participants that made choices based on the offer currently presented on the screen and the search value derived from the optimal decision-tree model (figure S1) and different other additions, described below. We then performed the same regression analyses on the simulated data from the different models as we did on the data of the real participants. F) We validated the sensitivity of bGLM1b (figure S2), i.e. the regression analysis to show that participants used a prospective value derived from a decision-tree search, rather than using a simple heuristic not requiring mental simulation. We compared a model that used the search value from the optimal decision-tree model ('Prospective model'), with a model that instead derived the search value as the best possible linear approximation ('Approximation only') given the myopic value, the number of available searches, the standard deviation of the patches in the environment (STD), the cost of searching and the interactions: STD x number of available searches and STD x cost of searching. The weights for each factor for the best linear approximation were derived by entering all searches across all participants into a linear regression predicting value of searching by the linear factors. Given these weights and the values for each of the constituting factors on each search in each trial, we then computed an approximation to the value of searching for each trial that did not rely on simulating a decision tree. We found that bGLM1b was sensitive: it showed a significant effect of (orthogonalized) prospective value beyond the linear components for the 'Prospective model', but does not find an effect for the 'Approximation only' model. G) validated the sensitivity of bGLM3a (figures 2E), i.e. analyses that tested whether participants adapted their perceived prospective value across searches in the trial appropriately or instead (wrongly) continued using the prospective value from the initial search. For this we simulated models that either used the prospective value of the initial search at each subsequent search ('ProspStatic') or that adapted correctly and used the currently correct prospective value ('PropsVAdapted') or a model that was half-way in between ('ProspVMixed'). We also simulated a model that fully adapted its prospective value, but had a bias to search more the more it had already searched ('Bias #Prev Searches'). We found that indeed bGLM3a showed the expected results: when controlling for the prospective value of the initial search, models that used the adjusted (i.e. decreased) prospective value of the current search showed a negative regression weight for the regressor capturing the difference between the initial and the current search's prospective value. H) We validated the sensitivity of bGLM5a, i.e. the analyses that investigated whether participants were less likely to search on the first search if the prospective value might decrease strongly over subsequent searches. We simulated data from a model whose decisions were only driven by prospective value, offer, cost and myopic value ('standard model') and from a model whose decisions were also driven by how much the prospective value might decrease per search ('pre-emptive adjustment' model). Again we found that only the 'pre-emptive', but not the 'standard' model showed the expected effect. This was particularly reassuring given that the regressors for prospective value and change in prospective value were highly correlated (Fig. S5D).*

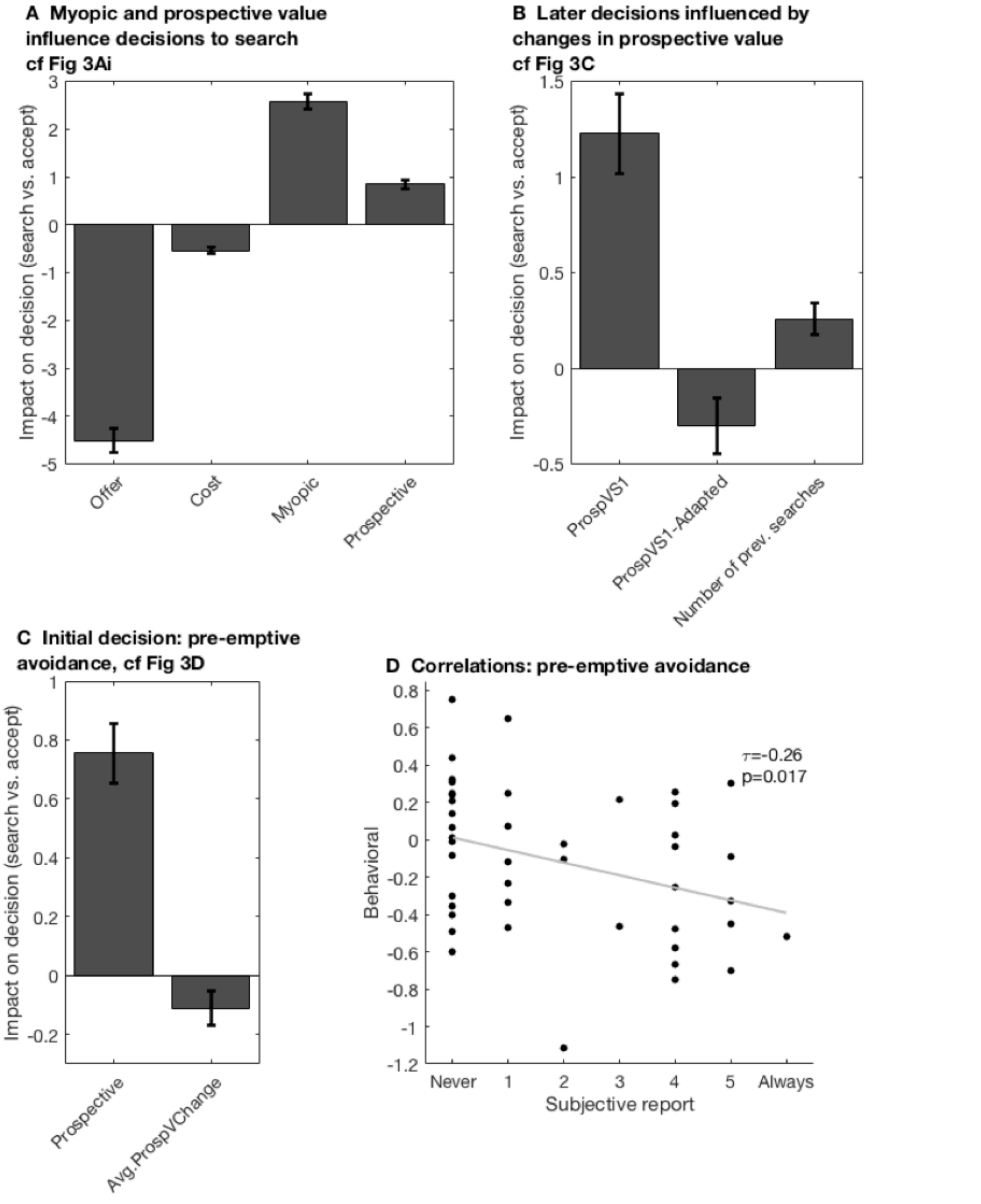

Figure S3. Data from the online sample, Related to Figure 4. *To verify that behavior was comparable in the online sample (n=51), we repeated the key analyses that had been conducted for participants in the*

FMRI scanning experiment (using one-tailed t-tests as we wanted to test whether the effect was in the same direction as in the fMRI sample). Overall, we found that participants' behavior was somewhat noisier; for example, for some participants not all regression analyses could be performed (because of too little variance in their choices, e.g. some participants almost always chose to search at the first decision on each trial) or some participants sometimes showed outlier regression weights (defined as more extreme than group mean ± 3 standard deviations). In these cases, their data was removed for those analyses. However, despite this, the pattern of results was very similar. A) Analyzing all searches together (analogous to Fig 3Ai), we found that participants were sensitive to all task relevant factors (all $p<10^{-20}$, n=51). B) Analyzing only later searches (i.e. excluding the first search on each trial – analogous to figure 3C), we found that participants adjusted their prospective value estimates as predicted by the model (regression weight for the change in prospective value ['ProspVS1-Adapted']: Wilcoxon signed rank test: z=-3.2, p= 0.00056, n=50). At the same time, participants also again showed a bias to over-persevere (regression weight for the number of previous searches done on this trial ['Number of prev. searches']: Wilcoxon signed rank test: z=2.9, p=0.0017, n=50). C) Examining the pre-emptive avoidance on the first search on each trial (analogous to figure 3D), we found that participants again showed this effect, though somewhat less than in the FMRI sample (t(46)=-1.94, p=0.029, n=47). The pre-emptive bias was potentially reduced because of larger individual differences in the online sample that had only been pre-screened for age-range, but was otherwise not selected to be mostly highly educated students (as had been the case in the FMRI sample); additionally, as they performed the task over the internet from their homes, there was no control over how much they might have been distracted. D) In support of the notion that individual differences were responsible for the reduced size of the pre-emptive avoidance effect, we found – in addition to the moderation effect noted in the main manuscript, figure 4E) – that pre-emptive avoidance measured behaviorally (Avg.ProspVChange regressor) was stronger (i.e. more negative regression weight) in participants who also reported in the debrief questionnaire (Q13, see methods and figure 4C) that they used this strategy (Non-parametric correlation, Kendall's tau= -0.26, p=0.017, n=47, two-tailed test as analysis had not been included in the FMRI sample). Possibly, if we had collected the debrief questionnaire in the FMRI sample, we might have found that they as a group reported higher scores.

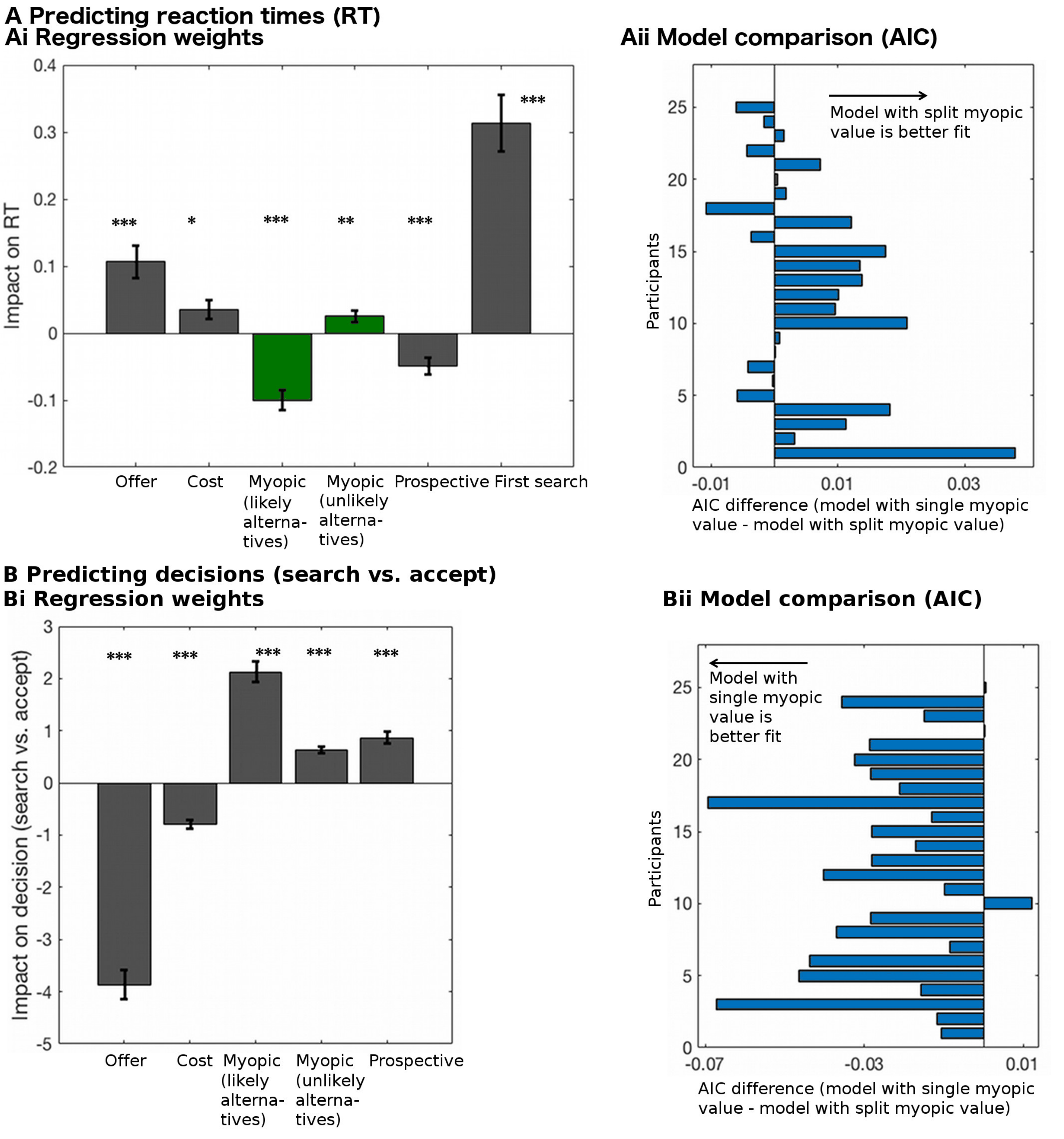

Figure S4: Reaction times (RTs) show that myopic utility is not unitary. Related to Figure 5. *A) We ran a regression analysis (bGLM1c, see also Methods section in main text: 'Analysis of all decisions') on RTs from all choices to test whether, for the fMRI analyses, myopic value should be included as a single regressor (as done for the behavioral analyses in the main text) or should be separated out into two components Ai) We found that while the myopic value of the three most likely alternatives was associated with decreased RTs (t(24)=-6.73, p=6*10^{-7}), the myopic value of the less likely alternatives increased RTs (t(24)=3.18, p=0.004). Aii) Similarly, model-comparison based on average Akaike information criterion (AIC) values per search, showed that for most participants (17 out of 25) and for the group as a whole, a model in which myopic value is split into separate components explained RTs better (summed AIC difference = 50). Given that values of the less likely alternatives slowed participants down, we decomposed myopic value into two separate component regressors also in the fMRI analyses. This is because neural signals related to the more unlikely components of myopic value might have a different and delayed timecourse compared to the more*

*likely components B) We performed the same regression analyses on participants' choices to search or accept. In contrast to RTs, we did not find evidence that a decomposition of myopic value into two component parts explained behavior better than a single measure of myopic value (Bii): model comparison (with AIC) indicated that for 22 out of 25 participants a model with a single myopic value fits the data better (summed AIC difference: 231). For this reason, in the analyses of decisions in the main text, we included myopic value as a single regressor. Overall, these analyses suggest that while participants use a myopic value estimate approximately resembling the average value all alternatives when making decisions, the onset and timing of neural signals relating to different component aspects of myopic value might vary. We thus modeled both parts of myopic value separately in the fMRI analysis. *p<0.05;**p<0.01;***p<0.001.*

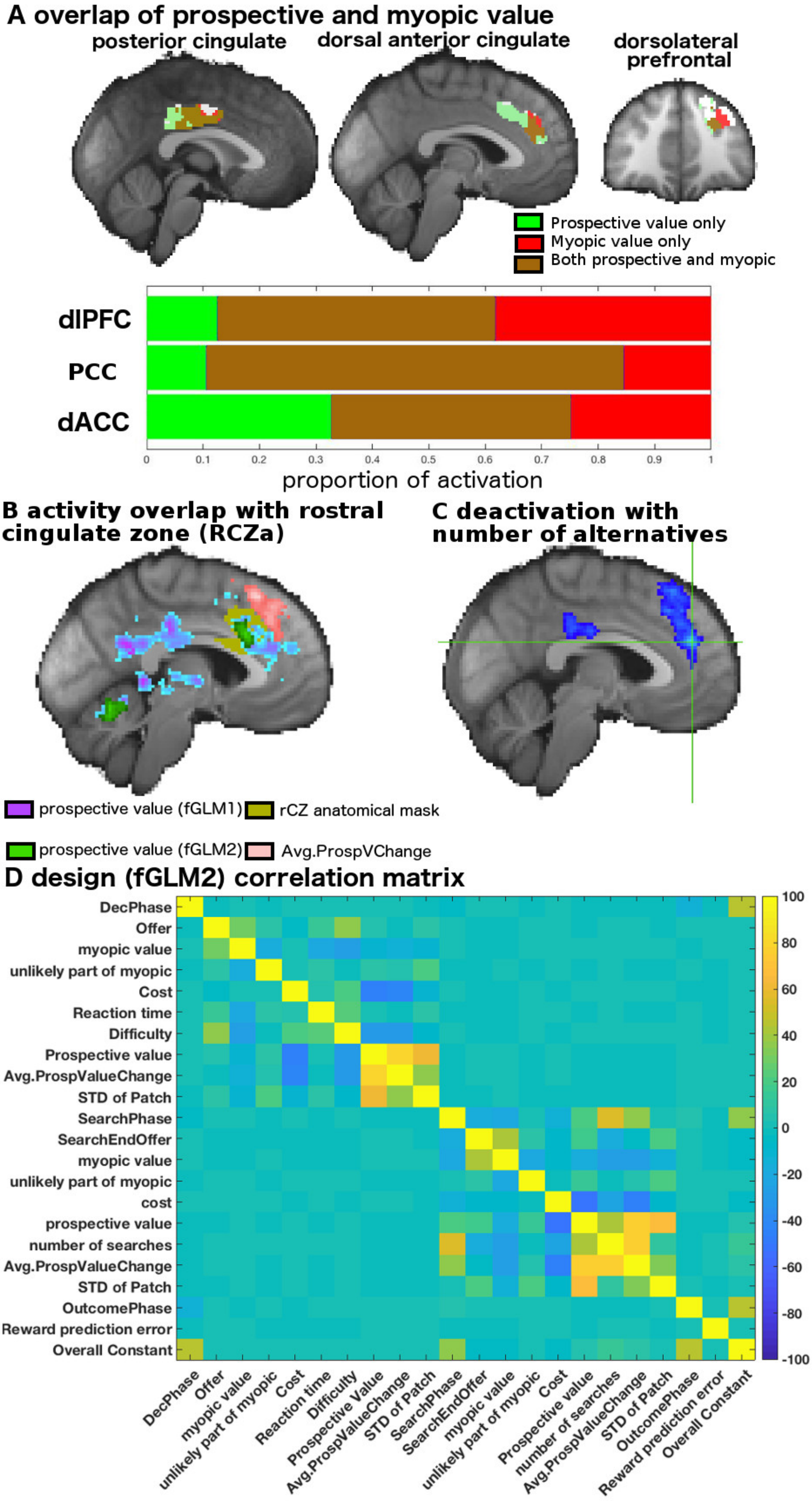
A overlap of prospective and myopic value
posterior cingulate
dorsal anterior cingulate
dorsolateral prefrontal
Prospective value only
Myopic value only
Both prospective and myopic
dlPFC
PCC
dACC
0
0.1
0.2
0.3
0.4
0.5
0.6
0.7
0.8
0.9
1
proportion of activation
B activity overlap with rostral cingulate zone (RCZa)
C deactivation with number of alternatives
prospective value (fGLM1)
rCZ anatomical mask
prospective value (fGLM2)
Avg.ProspVChange
D design (fGLM2) correlation matrix
DecPhase
Offer
myopic value
unlikely part of myopic
Cost
Reaction time
Difficulty
Prospective value
Avg.ProspValueChange
STD of Patch
SearchPhase
SearchEndOffer
myopic value
unlikely part of myopic
cost
prospective value
number of searches
Avg.ProspValueChange
STD of Patch
OutcomePhase
Reward prediction error
Overall Constant
100
80
60
40
20
0
-20
-40
-60
-80
-100

Figure S5. Overlap of neural signals for prospective and myopic value and fMRI design matrix. Related to Figure 5. ***A)*** *Illustration of the overlap between myopic and prospective value in three different anatomical areas (see methods section). Posterior cingulate (PCC), dorsal anterior cingulate (dACC) and dorsolateral prefrontal cortex (dlPFC) all contain regions in which prospective value and myopic value signals overlap (copper), as well as regions in which only prospective value (green) or myopic value (red) appear within each anatomically defined brain region (white) based on the atlases of Sallet and colleagues (Sallet et al., 2013) and Neubert and colleagues (2015); specifically, PCC included the anatomical Brodmann area 23ab (note that posterior boundary of the PCC region is an arbitrary consequence of the experimenters' initial decision of which areas to investigate – this was the most posterior region identified); dACC included the rostral cingulate zone and Brodmann area 32d; dlPFC included Brodmann areas 8B and 9/46d. Bars illustrate the percentage of voxels in each region that were sensitive to prospective value only, myopic value only or both (relative to the total number of voxels sensitive to either contrast [mathematically: prospective ∪ myopic)]); see also table S2. B) Overlap of activations in dorsomedial prefrontal cortex. Prospective value is present in the anterior rostral cingulate zone (RCZa; yellow) of dACC in the simple design (fMRI GLM 1; blue-purple color) and in the design controlling for change in prospective value (fMRI GLM 2; green). In contrast, the signal related to possible future change in prospective value (Avg.ProspVChange; pink) is only found in dorsomedial frontal areas 8/9m. Results are cluster-corrected (p<0.05). C) As shown in fig 5Di, choice uncertainty, a measure of difficulty, did not activate dACC. Similarly, we show here that the number of alternatives in the environment did not activate dACC and in contrast in fact led to widespread deactivation in dACC and elsewhere. Results are cluster-corrected (p<0.05). D) Correlational matrix of regressors from fMRI design* fGLM2*. fGLM2 only differs from fGLM1 in the inclusion of the Avg.ProspValueChange regressor.*

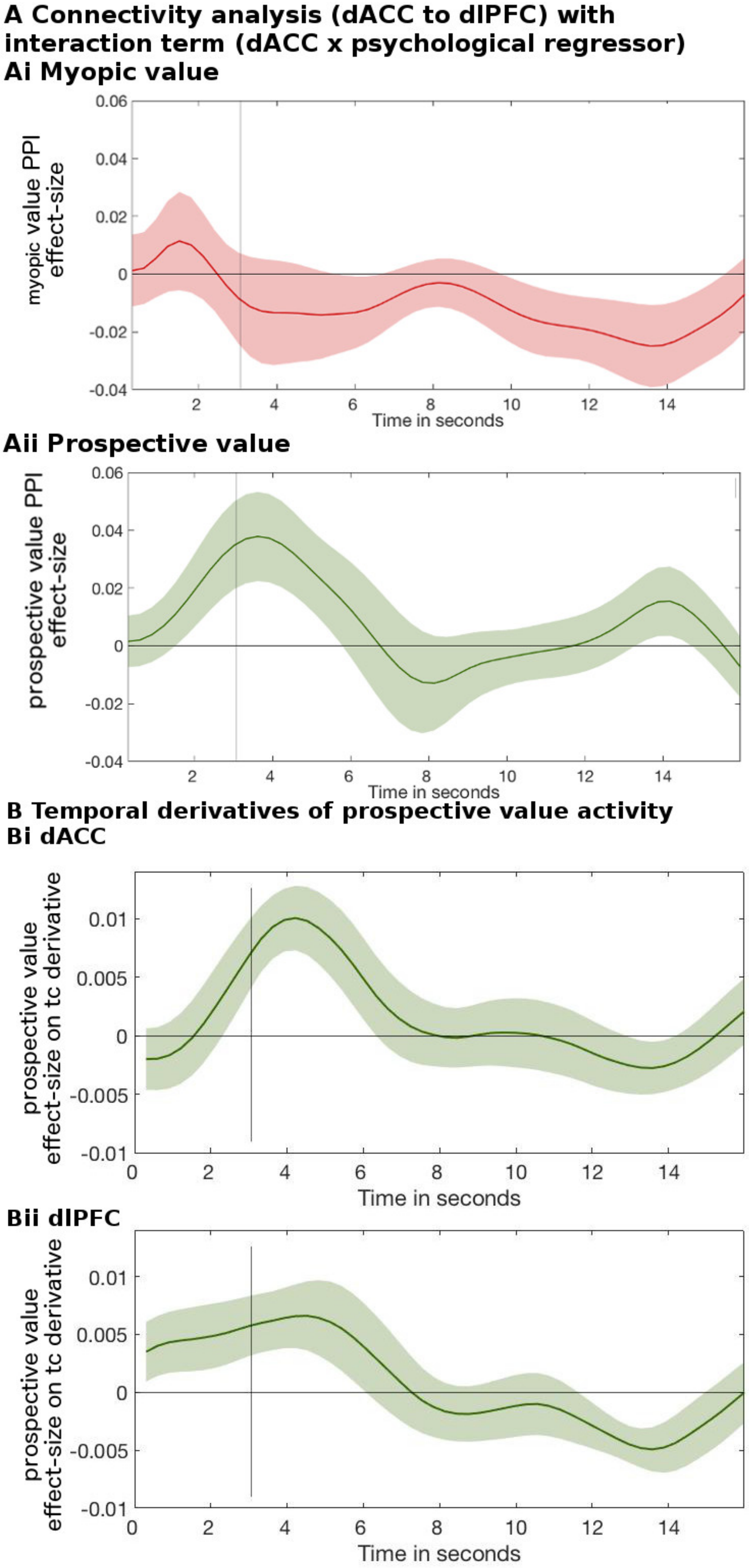

Figure S6. Control analyses for connectivity analyses and temporal derivatives of neural signal time courses. Related to Figure 5. ***A)*** *In the main text (fig. 5C) we computed psychophysiological interactions by splitting neural activity in the seed region by the psychological regressors and including these separately in a regression (e.g. predicting activity in dlPFC [target region] and including as separate regressors dACC [seed region] when prospective value was high or low, fig. 5Ci). An alternative and perhaps more common approach, albeit very similar in nature, is to compute the PPI by including an interaction term between the*

brain activity in the seed region and the psychological regressor. This is done here. We find the same results as before when looking at connectivity changes between dlPFC and dACC as a function of prospective value (Ai) or myopic value (Aii). B) We noted in the main text (fig. 5C) that the change in connectivity between dACC and dlPFC with prospective value occurs earlier than the onset of the brain signals related to prospective value per se*. In fact, this time point coincides with the period of fastest rise in brain activity in response to prospective value in both dACC (Bi) and dlPFC (Bii), as revealed by a regression analogous to those performed in fig. 5B, with the only difference being that the temporal derivative of the brain signal, rather than the brain signal itself is being predicted. Black vertical bars show the average response time for all participants.*

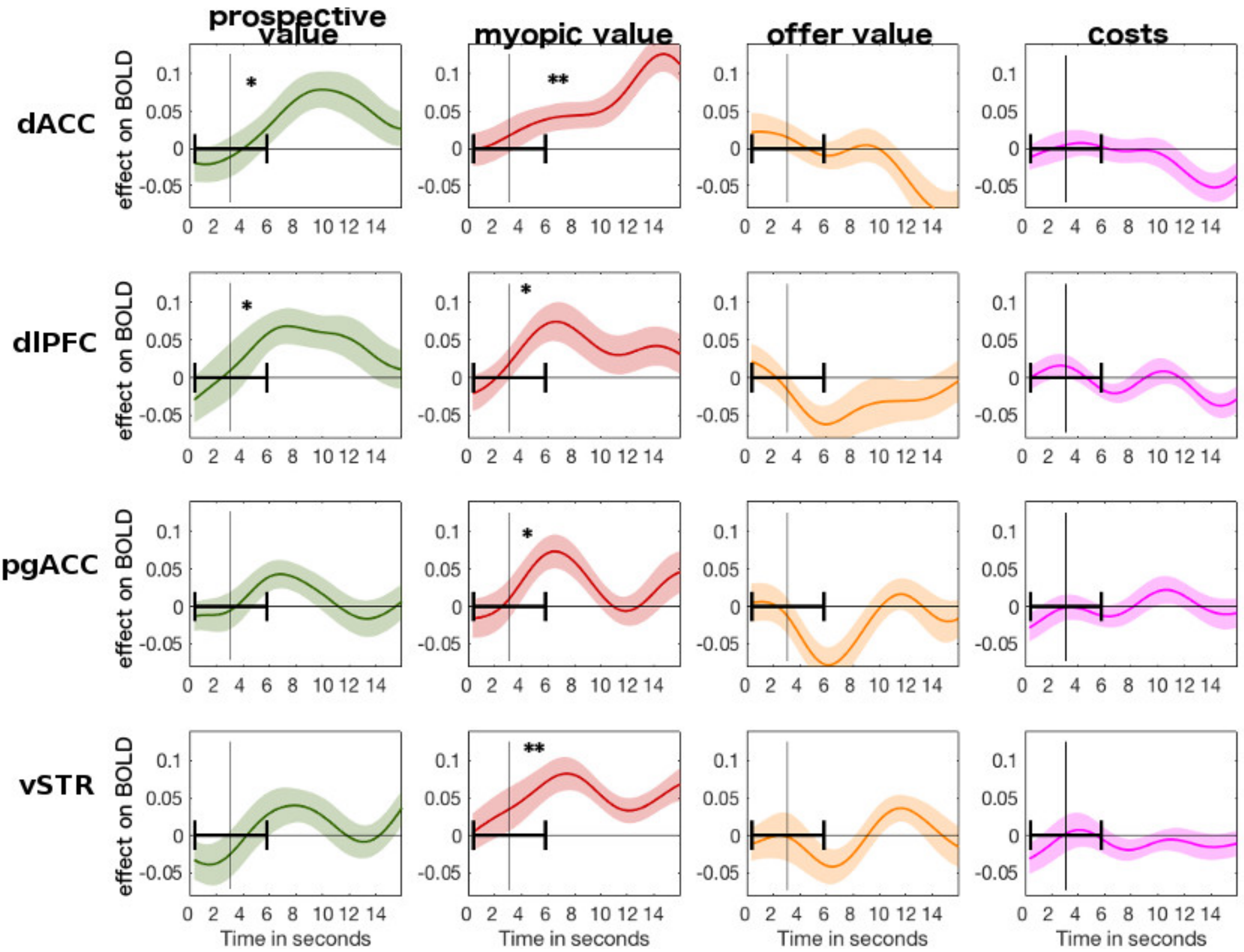

Figure S7. Time courses of neural activity related to different value and cost signals. Related to Figure 5. *Time courses of impact of different regressors on brain activity (BOLD) in different ROIs: dorsal anterior cingulate cortex (dACC), dorsolateral prefrontal cortex (dlPFC), perigenual anterior cingulate cortex (pgACC) and ventral striatum (vSTR). Data is shown as mean and standard error. Vertical lines show the average RT and the range of RTs is indicated by the black horizontal line. Significance stars show the results of significance tests conducted on the hemodynamically convolved peak activity (comparing it against zero), from leave-one-subject-out analyses (see Methods, section 'ROI timecourse and inter-regional activity correlation analyses') for details. *<0.05, **<0.01*

**Analyses done on all searches in each trial**									
bGLM1a	Constant	Offer value	Cost	Myopic value	Prospective value				
bGLM1b	Constant	Offer value	Cost	Myopic value	Prospective value (orthogonalized)	# Available searches	Standard deviation of patches (STD)	# Available searches x STD	# Available searches x cost
bGLM1b2	Constant	Offer value	Cost	Myopic value		# Available searches	Standard deviation of patches (STD)	# Available searches x STD	# Available searches x cost
bGLM1c	Constant	Offer value	Cost	Myopic value (3 most likely)	Myopic value (remaining)	Prospective value			
bGLM1d	Constant	Offer is highest and not last search	Offer is lowest and last search						
bGLM1e	Constant	Offer value	Cost	Myopic value	Highest available magnitude	Magnitude range			
bGLM1e2	Constant	Offer value	Cost	Myopic value	Highest available magnitude	Magnitude range	Prospective value		
**Analyses done on the later searches(≥2)**									
bGLM2a	Constant	Offer value	Search Value						
bGLM3a	Constant	Offer value	Cost	Myopic value	Prospective value at initial search	Prospective value change (initial-current)	# Previous searches in current trial		
bGLM4a (regressors as bGLM1a)	Constant	Offer value	Cost	Myopic value	Prospective value				
bGLM4b	Constant	Offer value	Cost	Myopic value	Approx. to prospective value (expo. decay)				
**Analysis done on initial search only**									
bGLM5a	Constant	Offer value	Cost	Myopic value	Prospective value	Average prospective value change			
bGLM5b (regressors as bGLM1a)	Constant	Offer value	Cost	Myopic value	Prospective value				

Table S1. List of regressors in each behavioral regression analysis*. Related to STAR methods. When analyses were run on reaction time (RT) data, rather than binary decisions, an additional regressor was included that specified whether a search was the first one on a given trial.*

	Threshold: p<0.05			Threshold: p<0.01		
	Myopic value	Prospective value	Myopic and prospective	Myopic value	Prospective value	Myopic and prospective
dACC	57%	75%	47%	27%	43%	25%
PCC	80%	85%	82%	50%	67%	57%
dlPFC	74%	62%	57%	39%	25%	22%

Table S2. Overlap of prospective and myopic value signals. Related to Figure 5, *Percentage of voxels in dorsal anterior cingulate cortex (dACC, comprising rostral cingulate zone and Brodmann area 32d), posterior cingulate cortex (PCC, Brodmann area 23ab) and dorsolateral prefrontal cortex (dlPFC, Brodmann areas 8b and 9/46d) that were sensitive to myopic value, prospective value or to both individually (=number of voxels sensitive to both contrasts divided by the number of voxels sensitive to either contrast, (prospective ∩ myopic)/ (prospective ∪ myopic)). Analyses were done separately at uncorrected thresholds p<0.05 and p<0.01*
